# Discovery of a class of glycosaminoglycan lyases with ultrabroad substrate spectrum and their substrate structure preferences

**DOI:** 10.1016/j.jbc.2024.107466

**Published:** 2024-06-12

**Authors:** Lin Wei, Ruyi Zou, Min Du, Qingdong Zhang, Danrong Lu, Yingying Xu, Xiangyu Xu, Wenshuang Wang, Yu-Zhong Zhang, Fuchuan Li

**Affiliations:** 1 National Glycoengineering Research Center and Shandong Key Laboratory of Carbohydrate Chemistry and Glycobiology, Shandong University, Qingdao, China; 2 School of Life Science and Technology, Weifang Medical University, Weifang, China; 3 MOE Key Laboratory of Evolution and Marine Biodiversity, Frontiers Science Center for Deep Ocean Multispheres and Earth System & College of Marine Life Sciences, Ocean University of China, Qingdao, China; 4 Marine Biotechnology Research Center, State Key Laboratory of Microbial Technology, Shandong University, Qingdao, China; 5 Joint Research Center for Marine Microbial Science and Technology, Shandong University and Ocean University of China, Qingdao, China

**Keywords:** polysaccharide lyase, polysaccharide lyase 35 family, glycosaminoglycan lyase, substrate specificity, enzymatic property

## Abstract

Glycosaminoglycan (GAG) lyases are often strictly substrate specific, and it is especially difficult to simultaneously degrade GAGs with different types of glycosidic bonds. Herein, we found a new class of GAG lyases (GAGases) from different bacteria. These GAGases belong to polysaccharide lyase 35 family and share quite low homology with the identified GAG lyases. The most surprising thing is that GAGases can not only degrade three types of GAGs: hyaluronan, chondroitin sulfate, and heparan sulfate but also even one of them can also degrade alginate. Further investigation of structural preferences revealed that GAGases selectively act on GAG domains composed of non/6-*O*-/*N*-sulfated hexosamines and d-glucoronic acids as well as on alginate domains composed of d-mannuronic acids. In addition, GAG lyases were once speculated to have evolved from alginate lyases, but no transitional enzymes have been found. The discovery of GAGases not only broadens the category of GAG lyases, provides new enzymatic tools for the structural and functional studies of GAGs with specific structures, but also provides candidates for the evolution of GAG lyases.

Glycosaminoglycans (GAGs), a class of linear polyanionic polysaccharides composed of repeating hexosamine-containing disaccharides, are ubiquitously distributed on the cell surface, in intracellular compartments, and in the extracellular matrix of animal tissues (1). Based on the disaccharide composition and the type of glycosidic bonds between disaccharides, GAGs can be categorized into the following classes: hyaluronan (HA), chondroitin sulfate (CS)/dermatan sulfate (DS), keratan sulfate, and heparan sulfate (HS)/heparin (Hep). The abundance and structural properties of GAGs expressed in tissues are temporally and spatially specific, and GAGs with specific structures have been shown to interact with a series of proteins, such as chemokines (2, 3), growth factors (4, 5) or other extracellular matrix components, and thus participate in various physiological and pathological processes. Except for keratan sulfate, which does not contain hexuronic acid (HexUA) residues, HA, CS/DS, and Hep/HS are composed of repeating disaccharides that contain HexUA (d-glucuronic acid [GlcUA] or l-iduronic acid [IdoUA]) and hexosamine (GalNAc or GlcNAc). Among these classes, HA is the simplest nonsulfate GAG constituted by repeated connections of disaccharide GlcUAβ1–3GlcNAc *via* β1–4 glycosidic bonds. In contrast, CS/DS and Hep/HS chains are much more complex because of the sulfation and epimerization of saccharide residues during biosynthesis (1). The basic bone of the CS chain is composed of repeating disaccharide GlcUAβ1–3GalNAc linked through β1–4 glycosidic bonds and is further modified at C-2 of GlcUA–IdoUA and C-4 and C-6 of GalNAc by various sulfotransferases (6). Meanwhile, some GlcUA residues are epimerized into IdoUA by the action of glucuronyl C-5 epimerase. The chain formed by repeated disaccharides of -4IdoUA1–3GalNAc1- is designated DS, and thus CS and DS often occur as a hybrid structure CS–DS (1). Similar to the case of CS–DS, the HS–Hep precursor composed of disaccharide -4GlcUA1–4GlcN/GlcNAc1- is sulfated at C-2 of GlcUA, and C-3 and C-6 of GlcNAc residues can be deacetylated and subsequently sulfated to form GlcNS. In addition, GlcUA is also often epimerized to IdoUA, especially in Hep chains (1).

Polysaccharide lyases (PLs) catalyze the degradation of HexUA-containing polysaccharides employing a β-elimination reaction. Unlike eukaryotic-derived glycoside hydrolases that cleave uronic acid–containing polysaccharides through a hydrolysis mechanism, microbial-derived PLs do not require the participation of water molecules to degrade HexUA-containing polysaccharides but remove a molecule of water from the uronic acid residue being acted upon and introduce an unsaturated double bond to the C4 and C5 of the uronic acid residue at the newly generated reducing end (7). GAG lyases, a series of microorganism-derived enzymes belonging to the superfamily of PLs, specifically catalyze the degradation of HexUA-containing GAGs. The identified GAG lyases are widely distributed in the PL6, PL8, PL12, PL13, PL15, PL16, PL21, PL23, PL29, PL30, PL33, PL35, and PL37 families (8). All identified GAG lyases possess substrate specificity to strictly distinguish the saccharide composition and inside glycosidic bond of GAG chains. Based on substrate specificity, they are usually divided into the following categories: HA-specific lyases, which specifically digest HA (9, 10); CS/DS lyases, which are traditionally called chondroitinases (CSases), and can degrade CS/DS as well as HA in most cases (11, 12, 13); and Hep/HS lyases (hepases), which specifically cleave Hep/HS (14, 15, 16). To date, no GAG lyase has been discovered that can degrade three types of GAGs: HA, CS, and HS.

In this study, a new class of PLs belonging to the PL35 family was identified and named GAGases. In contrast to the identified GAG lyases, which exhibit strict substrate specificity to either HA/CS/DS or Hep/HS, GAGases can simultaneously degrade three types of GAGs (HA, CS, and HS), and one of them can even efficiently cleave alginate, a completely different HexUA-containing polysaccharide, which is composed of d-mannuronate (M) and its C5 epimer L-guluronate (G), and M and G residues often alternate randomly to form heteropolyuronic blocks. The basic biochemical characteristics and substrate-structure preferences of these GAGases were studied in detail. The discovery of GAGases with ultrabroad substrate spectrum enriches the library of GAG lyases, provides new enzymatic tools for the structural and functional studies of GAGs with specific structures, and may also help reveal the evolution of GAG lyases.

## Results

### Sequence properties of GAGases belonging to the PL35 family

**Figure 1:**
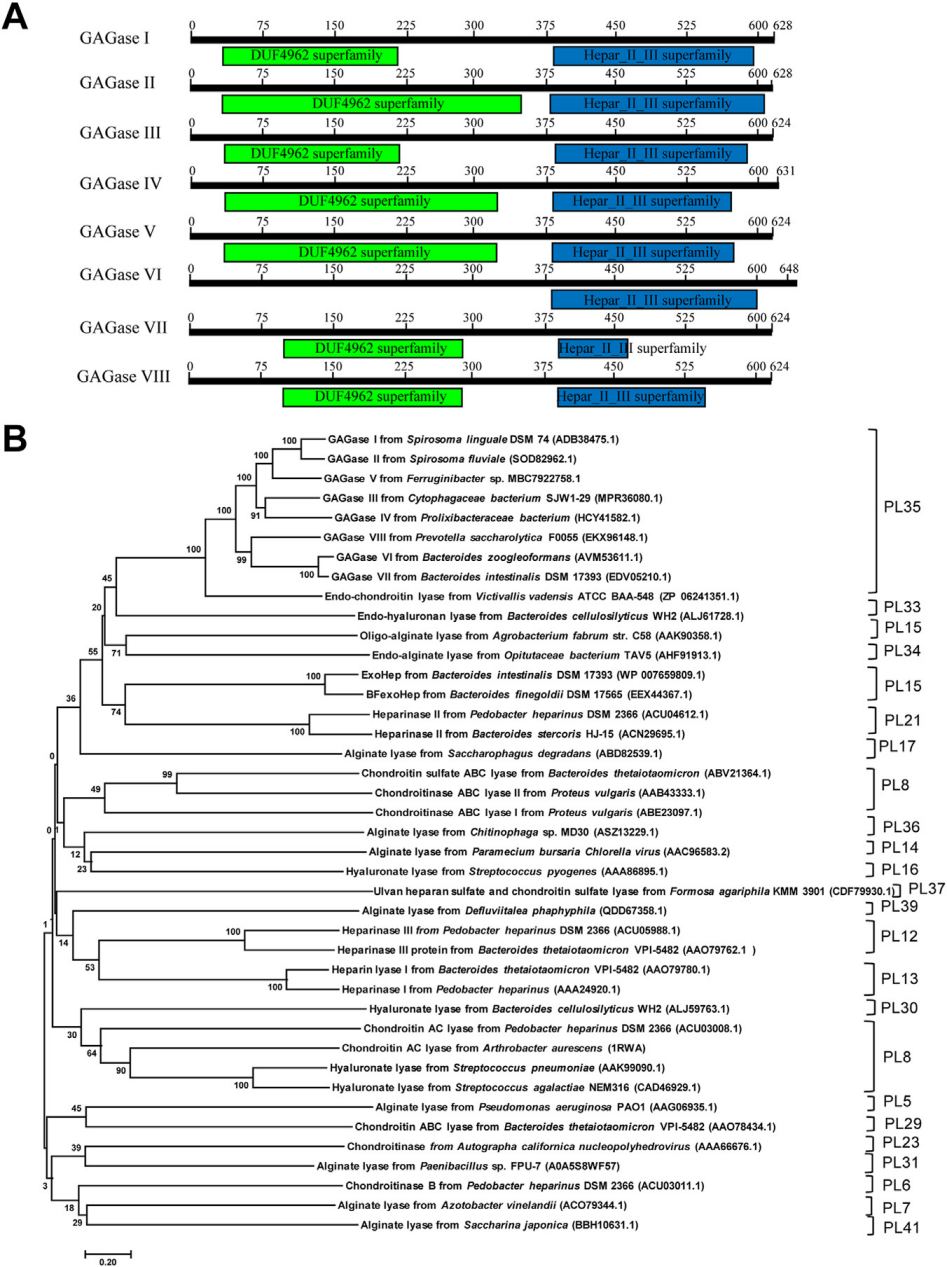
**Functional modules and phylogenetic analysis of GAGases.***A*, functional modules of PL35 family GAGases. *B*, phylogenetic analyses of GAGases I–VIII with identified GAG lyases and alginate lyases were executed based on Clustal W multiple alignments with identified GAGs and alginate lyases from bacteria. Phylogenetic tree was built with MEGA, version 7.0.26, software. Evolutionary history was inferred using the neighbor-joining method. The optimal tree with the sum of branch length of 18.94 is shown. All positions with less than 50% site coverage were eliminated. The percentage of replicate trees in which the associated taxa clustered together in the bootstrap test (998 replicates) is shown next to the branches. GAG, glycosaminoglycan; GAGase, GAG lyase.

While searching for novel GAG lyases, we noticed a putative gene (*GAGase I*) from *Spirosoma linguale* DSM 74, which is assigned as heparinase II/III in GenBank but shares very low similarity with all identified GAG lyases, including various heparinases. Interestingly, a series of homologous sequences of GAGase I have been found in the gene database, and all these sequences (GAGases I–VIII) contain approximately 1800 bases that encode ∼70 kDa proteins containing 624 to 648 amino acid residues (Table 1). In addition, the sequences consist of an N-terminal “DUF4962 superfamily” module and a C-terminal “Hepar_II_III superfamily” module, which is similar to the case of PL15 members, except for GAGase VI without the typical N-terminal module (Fig. 1*A*). Furthermore, phylogenetic analysis revealed that these unidentified sequences were distant from various identified GAG lyases and clustered independently into the PL35 family (Fig. 1*B*). In comparison, these potential GAG lyases not only share the highest homology (identity of 35–37% with 69%-77% query coverage) with an endo-CSase in the PL35 family but are also relatively close to an endo-HA lyase in the PL33 family (17), an alginate lyase and an exoHepase in the PL15 family (18, 19), two heparinase II in the PL21 family (20, 21), and an alginate lyase in the PL34 family (17) (Fig. 1*B*). Altogether, these unique sequence properties suggest the potential novelty of these GAGases because of their enzymatic properties.

**Table 1:** Gene and protein sequence information of GAGases

Name	GenBank accession number	Total residue	Gene length (bp)	Molecular mass (kDa)	Isoelectric point	Signal peptide	Sequence identity (query cover/percent identity)^a^
GAGase I	ADB38475.1	628	1818	70.1	8.41	Sec/SPI^b^ (Met^1^–Ala^23^)	100%/100%
GAGase II	SOD82962.1	628	1815	69.9	6.54	Sec/SPI (Met^1^–Ala^23^)	100%/81.2%
GAGase III	MBC7922758.1	624	1809	69.2	7.67	Sec/SPI (Met^1^–Ala^21^)	98%/66.1%
GAGase IV	MPR36080.1	631	1806	71.0	6.75	Sec/SPI (Met^1^–Ala^29^)	98%/55.0%
GAGase V	HCY41582.1	624	1806	70.1	7.15	Sec/SPI (Met^1^–Ala^22^)	99%/55.5%
GAGase VI	EKX96148.1	648	1818	72.9	9.48	None^c^	99%/49.6%
GAGase VII	EDV05210.1	624	1827	70.5	5.69	Sec/SPII (Met^1^–Ala^15^)	93%/44.8%
GAGase VIII	AVM53611.1	624	1815	71.0	6.41	Sec/SPII^d^ (Met^1^–Ala^15^)	93%/42.5%

a Sequence identity means the sequence similarity compared with GAGase I.

b Sec/SPI: “standard” secretory signal peptides transported by the Sec translocon and cleaved by signal peptidase I (Lep).

c None: do not have any predicted signal peptide.

d Sec/SPII: lipoprotein signal peptides transported by the Sec translocon and cleaved by signal peptidase II (Lsp).

### Heterologous expression and purification of GAGases

**Figure 2:**
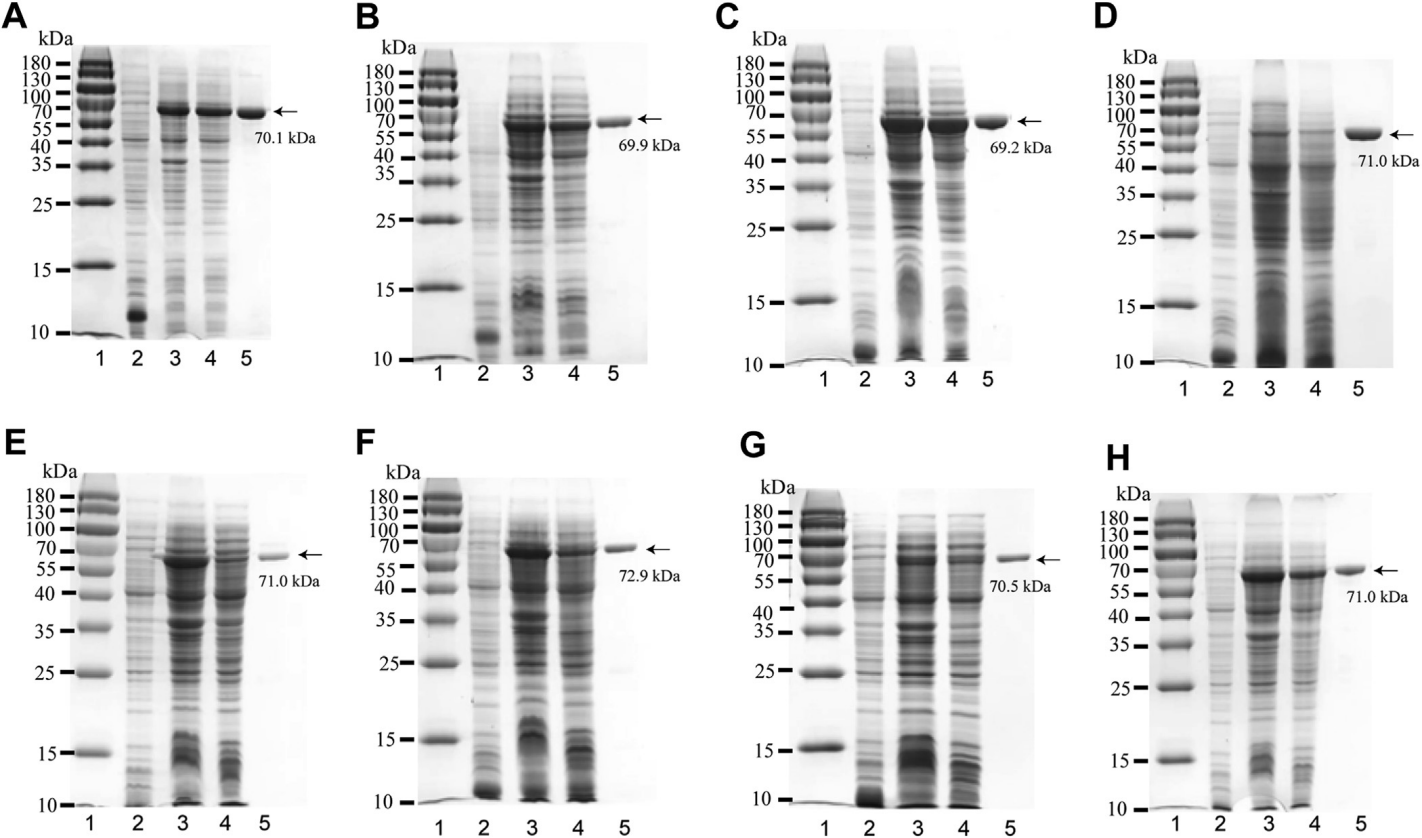
**Expression and purification of recombinant GAGases.** GAGase I–VIII (*A*–*H*) were purified and assessed by SDS-PAGE using 13.2% polyacrylamide gel, followed by staining with Coomassie brilliant blue. Lane 1, 180 kDa Prestained Protein Marker (MP102) (Vazyme); lane 2, lysate of cells transfected with empty plasmid (pET-30a); lane 3, lysate of IPTG-induced cells transfected with expression plasmid; lane 4, lysate supernatant of IPTG-induced cells transfected with expression plasmid; and lane 5, purified recombinant enzymes with Ni^2+^ chelation chromatography. GAG, glycosaminoglycan; GAGase, GAG lyase.

The constructed pET30a-GAGases were transferred into *Escherichia coli* BL21 (DE3) cells. The cells harboring the expression vectors were cultured and induced by IPTG. The recombinant proteins in supernatants were extracted from the pET30a-GAGase-harboring host cells by sonication and centrifugation and purified using nickel–nitrilotriacetic acid affinity chromatography. According to the results of SDS-PAGE analysis, the recombinant GAGase proteins were successfully expressed as soluble forms with a molecular mass of about 70 kDa and could be purified to a purity of about 95% by a single nickel affinity chromatography (Fig. 2).

### Substrate specificities of GAGases

**Figure 3:**
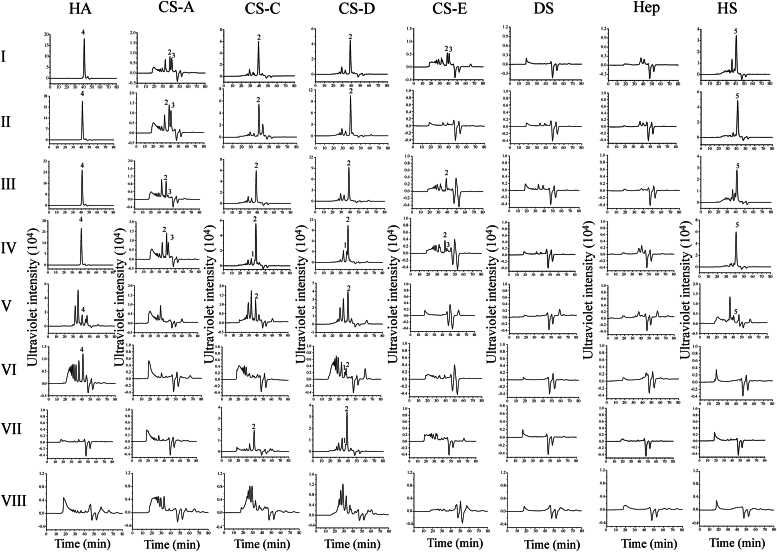
**Product analysis of PL35 family GAGases.** GAGs (30 μg) were individually digested with GAGases (6 μg) at 30 °C and analyzed by gel filtration chromatography on Superdex Peptide column. The elution positions of unsaturated disaccharides are indicated as follows: 1, disulfated unsaturated CS disaccharides; 2, monosulfated unsaturated CS disaccharides; 3, nonsulfated unsaturated CS disaccharides; 4, nonsulfated unsaturated HA disaccharides; 5, monosulfated or nonsulfated unsaturated HS disaccharides. CS, chondroitin sulfate; GAG, glycosaminoglycan; GAGase, GAG lyase; HA, hyaluronan; HS, heparan sulfate.

To investigate the enzymatic properties of these potential GAG lyases, GAGases I–VIII were individually recombinantly expressed and purified as described in the “*Experimental procedures*” section. The specificity of each GAGase was evaluated by using various GAGs as substrates (Table S1). Surprisingly, analysis of the resulting reactants by gel filtration chromatography showed that GAGase I–V could efficiently digest not only HA and various CS variants but also HS, even GAGase III could significantly degrade alginate, which is composed of d-mannuronate (M) and its C5 epimer l-guluronate (G) and alternate randomly to form heteropolyuronic blocks (Figs. 3 and S1), suggesting that these enzymes have ultrabroad substrate spectra distinct from the identified GAG lyases. In contrast, GAGase VI–VIII exhibited relatively weak and limited substrate degradability (Fig. 3). Notably, almost all these enzymes showed different abilities to degrade GlcUA-containing HA and various CSs but could not easily degrade IdoUA-rich DS and Hep. Furthermore, these enzymes appear to better degrade CS-C and CS-D, which are rich in 6-*O*-sulfated GalNAc residues, than CS-A and CS-E, which are rich in 4-*O*-sulfated GalNAc residues (Fig. 3 and Table S1).

**Figure 4:**
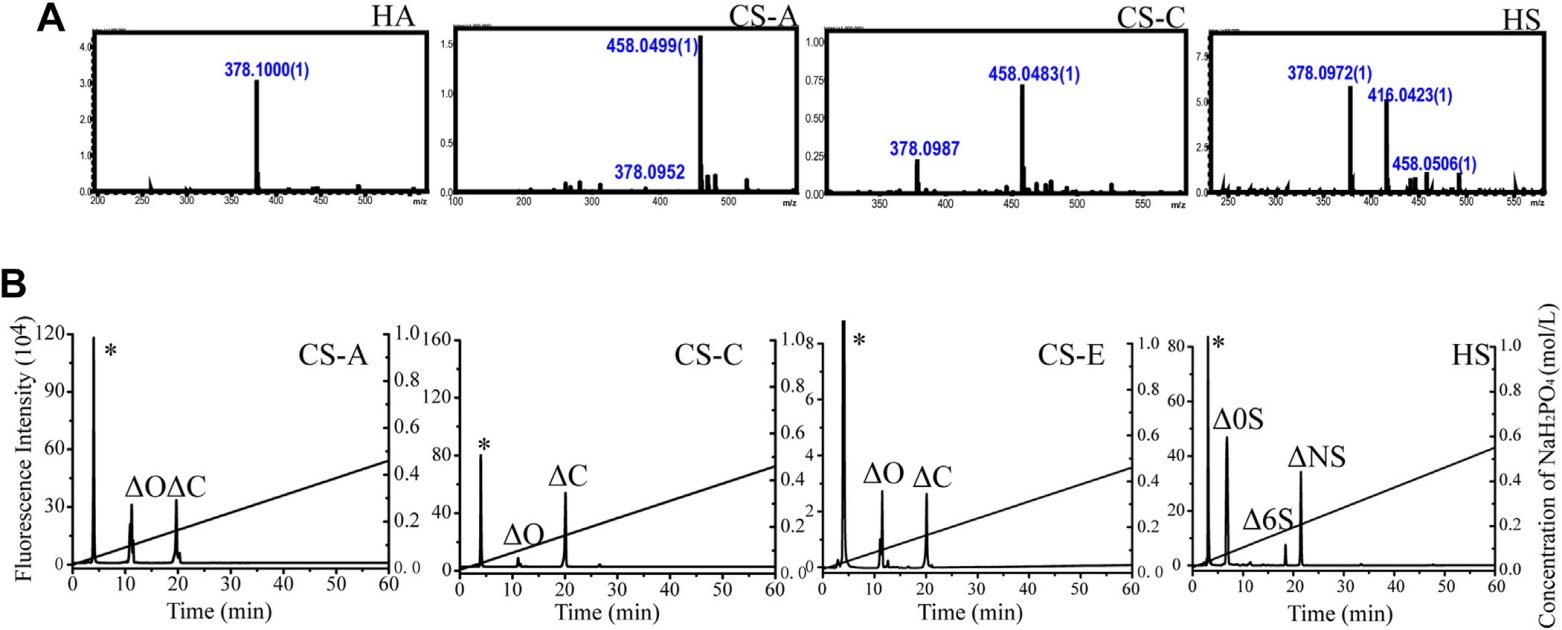
**Composition analysis of disaccharides in the final products of GAGs digested by GAGase I.** The disaccharide fractions in the final products of HA, CS-A, CS-C, and HS digested by GAGase I were collected and analyzed by liquid chromatography–ion trap time-of-flight hybrid mass spectrometry (*A*) and anion-exchange HPLC (*B*). The relevant signals are indicated as follows: ∗, the peaks detected around 3 min in the figures are derived from fluorescent labeling reagents; ΔO, Δ^4,5^HexUA1–3GalNAc, ΔC, Δ^4,5^HexUA1–3GalNAc(6S); Δ0S, Δ^4,5^HexUA1–4GlcNAc; Δ6S,Δ^4,5^HexUA1–4GlcNAc(6S); and ΔNS, Δ^4,5^HexUA1–4GlcNS. CS, chondroitin sulfate; GAG, glycosaminoglycan; GAGase, GAG lyase; HA, hyaluronan; HS, heparan sulfate.

To further reveal the preferred structures of these GAGases, the disaccharide fraction of each GAG digested with GAGase I was collected during the aforementioned gel filtration analysis (Fig. 3) and analyzed by liquid chromatography–ion trap time-of-flight hybrid mass spectrometry. Two major peaks at 378.10 *m*/*z* and 458.06 *m*/*z* were detected in the digests of HA/CS/HS and assigned to the unsaturated *N*-acetylated nonsulfated and monosulfated disaccharide, respectively, and a peak of 416.04 *m*/*z* in the digest of HS was assigned to the *N*-sulfated unsaturated disaccharide (Fig. 4*A*). Furthermore, disaccharide composition analysis by anion-exchange HPLC showed that the nonsulfated disaccharides were ΔHexUA1–3GalNAc (ΔO) from various CSs and ΔHexUA1–4GlcNAc (Δ0S) from HS, the monosulfated *N*-acetylated disaccharides were ΔHexUA1–3GalNAc(6S) (ΔC) from CSs and ΔHexUA1–4GlcNAc(6S) (Δ6S) from HS, and the *N*-sulfated disaccharide corresponded to ΔHexUA1–4GlcNS (ΔNS) from HS (Fig. 4*B*). These results indicate that, regardless of the structure of hexosamine and the type of glycosidic bond between the repeating disaccharide units in HA/CS/HS, GAGases prefer to act on the domains composed of non/6-*O*-/*N*-sulfated disaccharides. Moreover, GAGase I can digest GAGs extracted from mouse breast cancer 4T1 cells and normal human embryonic kidney 293T cells, but GAGase I prefers to GAGs from 4T1 cells to produce more nonsulfated disaccharides than GAGs from 293T cells (Fig. S2), indicating that GAGs from different types of cells differ significantly in their internal structures.

### Basic enzymatic characteristics of GAGases

The basic biochemical characteristics of these GAGases were investigated by using HA as a substrate, which has the simplest structure and is preferred by most of these enzymes. Except for a few cases, these enzymes have the same or similar optimum reaction temperature (40 °C) and optimum buffer (Tris–HCl, pH 7–8), and most of them are weakly stimulated by alkali metal ions Na^+^ and K^+^ but strongly inhibited by various heavy metal ions, such as Pb^2+^, Hg^2+^, Fe^3+^, and Cu^2+^ (Table 2). Notably, GAGase III and IV could be significantly stimulated by K^+^, which might be related to their origin from aquatic bacteria, and GAGase II, III, and IV were sensitive to stimulation by Ca^2+^, Mg^2+^, and Mn^2+^, especially GAGase III, which is strongly stimulated by Ca^2+^ and Mn^2+^ (Table 2). Under their respective optimal reaction conditions, the specific activities of these enzymes toward various substrates are not high, but their activities toward HA and CS are relatively high (Table 2), which may explain why they are usually regarded as HA–CS lyases. However, we found that these enzymes, such as GAGase I and IV, exhibit good temperature stability at 40 °C and below. This is especially true for GAGase I, as the enzyme exhibits temperature-stimulated activity and maintains high activity at its optimum temperature (20 °C) for more than a week (Fig. S3), which will compensate for its low specific activity in applications. Moreover, the apparent *K*_*m*_ and *V*_max_ values of GAGase I–V toward HA, CS-C, and HS were calculated, and the results showed that the apparent *K*_*m*_ values of them toward CS-C are obviously smaller than those toward HA and HS (Table 3), further confirming that GAGases prefer to act the highly 6-*O*-sulfated CS-C.

**Table 2:** The enzymatic properties of GAGases

Condition		GAGase I	GAGase II	GAGase III	GAGase IV	GAGase V	GAGase VI	GAGase VII	GAGase VIII
Optimal temperature (°C)		40	30	40	40	40	50	40	40
Optimal buffer (pH)		Tris–HCl (pH 7.0)	Tris–HCl (pH 7.0)	Tris–HCl (pH 7.0)	Tris–HCl (pH 7.0)	NaAc-HAc (pH 6.0)	Tris–HCl (pH 8.0)	NaAc-HAc (pH 6.0)	Tris–HCl (pH 8.0)
Effect of alkaline metal ions (5 mM)		KCl (104%)	KCl (104%)	KCl (120%)	KCl (139%)	NaCl (108%)	NaCl (113%)	ND	NaCl (107%)
				NaCl (109%)	NaCl (118%)				
Effect of metal cation (5 mM)	Enhancer	Mn^2+^ (107%)	Ca^2+^ (137%)	Ca^2+^ (173%)	Mg^2+^ (104%)	ND	ND	Ca^2+^ (102%)	Mn^2+^ (105%)
		Ba^2+^ (105%)	Mg^2+^ (145%)	Mg^2+^ (114%)	Mn^2+^ (126%)			Mn^2+^ (103%)	Ba^2+^ (108%)
			Mn^2+^ (116%)	Mn^2+^ (212%)					
	Inhibitor	Cr^3+^ (0%)	Pb^2+^ (0%)	Hg^2+^ (0%)	Fe^3+^ (0%)	Hg^2+^ (0%) Cu^2+^ (0%)	Cr^3+^ (0%)	Fe^3+^ (0%)	Fe^3+^ (0%)
		Pb^2+^ (0%)	Cu^2+^ (0%)	Fe^3+^ (0%)	Cr^3+^ (0%)	Zn^2+^ (0%)	Zn^2+^ (0%)	Cr^3+^ (0%)	Cr^3+^ (0%)
		Fe^2+^ (0%)	Zn^2+^ (0%)	Cu^2+^ (0%)	Pb^2+^ (0%)	Cr^3+^ (8%)		Pb^2+^ (0%)	Pb^2+^ (0%)
		Zn^2+^ (0%)	Cr^3+^ (4%)	Zn^2+^ (0%)	Cu^2+^ (4%)			Cu^2+^ (23%)	Cu^2+^ (0%)
		Cu^2+^ (6%)	Fe^3+^ (5%)	Pb^2+^ (3%)	Fe^2+^ (8%)			Zn^2+^ (28%)	Zn^2+^ (7%)
		Hg^2+^ (10%)	Hg^2+^ (9%)	Ni^2+^ (13%)	Zn^2+^ (12%)				Ni^2+^ (10%)
Effect of (5 mM) EDTA		60%	24%	78%	65%	88%	84%	73%	73%
Specific enzyme activity (U/mg)	HA	5.62 ± 0.22	2.81 ± 0.23	16.47 ± 1.03	13.63 ± 2.24	0.39 ± 0.04	0.10 ± 0.01	ND	ND
	CS-C	0.83 ± 0.25	0.62 ± 0.07	3.96 ± 0.22	5.61 ± 0.29	0.25 ± 0.01	0.13 ± 0.01	ND	ND
	HS	0.08 ± 0.05	0.28 ± 0.03	0.33 ± 0.09	0.71 ± 0.25	0.06 ± 0.04	ND	ND	ND
	Alginate	ND	ND	0.29 ± 0.07	ND	ND	ND	ND	ND

Abbreviation: ND, not detected.

**Table 3:** Apparent kinetic analysis of GAGase I–V toward HA, CS-C, HS, and alginate

Name	Substrate	*K*_*m*_ (mg/ml)	*V*_max_ (μmol/min/mg)
GAGase I	HA	5.08 ± 0.90	20.99 ± 2.41
	CS-C	1.67 ± 0.26	13.72 ± 0.94
	HS	2.91 ± 0.32	14.72 ± 0.87
GAGase II	HA	5.42 ± 2.05	4.02 ± 0.96
	CS-C	3.99 ± 1.58	2.85 ± 1.15
	HS	11.29 ± 5.78	11.01 ± 2.77
GAGase III	HA	4.34 ± 1.72	44.80 ± 11.05
	CS-C	3.37 ± 0.65	75.58 ± 8.27
	HS	6.60 ± 0.98	12.17 ± 3.86
	Alginate	8.05 ± 3.00	4.85 ± 1.434
GAGase IV	HA	12.52 ± 5.82	12.29 ± 4.62
	CS-C	4.46 ± 1.69	55.11 ± 13.08
	HS	5.68 ± 1.40	38.54 ± 6.38
GAGase V	HA	5.57 ± 2.87	1.11 ± 0.38
	CS-C	2.61 ± 0.31	3.09 ± 0.20
	HS	4.42 ± 1.04	1.67 ± 0.25

The K_*m*_ and *V*_max_ indicated the apparent K_*m*_ and *V*_max_.

To determine the action pattern of GAGases, HA was used as substrate and digested by GAGase I in a time-course experiment. According to the results showed in Fig. S4, GAGase I initially produces a series of large oligosaccharides, and then the size of the oligosaccharide products gradually decreases with increasing digestion time, indicating that GAGases are endo-type GAG lyases.

### Sulfation pattern preference of GAGases

**Figure 5:**
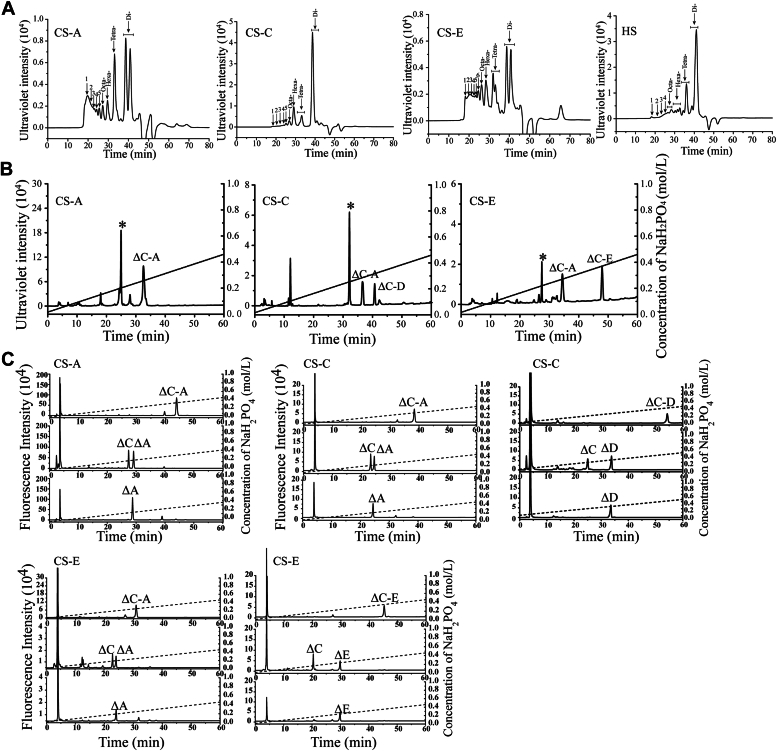
**Fractionation and major tetrasaccharide sequencing of the final products from the digestion of various GAGs by GAGase I.***A*, the CS-A, CS-C, CS-E, and HS were exhaustively degraded with GAGase I for 24 h, the final products were fractionated by gel filtration HPLC with detection at 232 nm, and the size-defined fractions were collected. The elution of each fraction is indicated as follows: 1, fraction 1; 2, fraction 2; 3, fraction 3; 4, fraction 4; 5, fraction 5; 6, fraction 6; Di-, disaccharide; Hexa-, hexasaccharide; Octa-, octasaccharide; and Tetra-, tetrasccharide. *B*, subfractionation of GAGase I-resistant CS tetrasaccharides. The tetrasaccharide fractions in the final products of CS-A, CS-C, and CS-E digested by GAGase I were further subfractionated by anion-exchange HPLC on a YMC-Pack PA-G column. *C*, sequencing of the purified tetrasaccharides. The purified tetrasaccharides from the final products of CS-A, CS-C, and CS-E digested by GAGase I were sequenced by using a enzymatic method as described in the “*Supporting methods*” section. *Top panel*, the tetrasaccharides were labeled with 2-AB; *middle panel*, the tetrasaccharides were labeled with 2-AB and then digested with HCDLase; *Bottom panel*, the CS tetrasaccharide was digested with CSase ABC and labeled with 2-AB. The relevant signals are indicated as follows: ∗, Component that cannot be identified; ΔO, Δ^4,5^HexUA1–3GalNAc; ΔC, Δ^4,5^HexUA1–3GalNAc(6S); ΔA, Δ^4,5^HexUA1–3GalNAc(4S); ΔD, Δ^4,5^HexUA(2S)1–3GalNAc(6S); and ΔE, Δ^4,5^HexUA1–3GalNAc(4S,6S). 2-AB, 2-aminobenzamide; CS, chondroitin sulfate; GAG, glycosaminoglycan; GAGase, GAG lyase; HS, heparan sulfate.

The aforementioned substrate specificity analysis preliminarily indicated that GAGases exhibit a preference for certain structures of substrates, including sulfation patterns and HexUA configurations. To further investigate the degrading capacities of GAGases toward different sulfation patterns, the disaccharide and resistant oligosaccharide fractions in the final products of various CSs and HS digested by GAGase I as a representative were collected and analyzed for their disaccharide compositions (Fig. 5*A* and Table S2). Similar to the results found in the digestion of CSs and HS by GAGase I, the disaccharide fractions in the final digests of these GAGs mainly contain non/6-*O*-/*N*-sulfated disaccharide units, whereas the contents of 4-*O*-, 2-*O*-, and high-sulfated disaccharide units increase as the polymerization degree of oligosaccharide fractions increases (Table S2), which further confirms that GAGases prefer to act on non/6-*O*-/*N*-sulfated disaccharide–contained domains but not other sulfation patterns such as A unit (GlcUA1–3GalNAc(4S)), D unit (GlcUA(2S)1–3GalNAc(6S)), or/and E unit (GlcUA1–3GalNAc(4S,6S))-contained CS domains and 2-*O*-/di/trisulfated disaccharide-contained HS domains.

To investigate the resistant structures of CS to the digestion by GAGases, the major tetrasaccharides in the final digests of CS variants CS-A, CS-C, and CS-E by GAGase I were purified (Fig. 5*A*) and sequenced as described in the “Experimental procedures” section. The results showed that these GAGase I-resistant tetrasaccharides in CS variants were ΔC-A (Δ^4,5^HexUA1-3GalNAc(6S)β1-4GlcUAβ1-3GalNAc(4S)), ΔC-D (Δ^4,5^HexUA1-3GalNAc(6S)β1-4GlcUA(2S)β1-3GalNAc(6S)), and ΔC-E (Δ^4,5^HexUA1-3GalNAc(6S)β1-4GlcUAβ1-3GalNAc(4S,6S)) (Fig. 5, *B* and *C*). Based on the structures of these resistant tetrasaccharides, it is very clear that the GAGase I-resistant tetrasaccharides in CS always have a degradable structure at the nonreducing end, such as 6-*O*-sulfated C unit, and a resistant structure at the reducing end, such as 4-*O*-sulfated A or E unit or 2-*O*-sulfated D unit, which is consistent with the results from the substrate specificity analysis of GAGase I.

**Figure 6:**
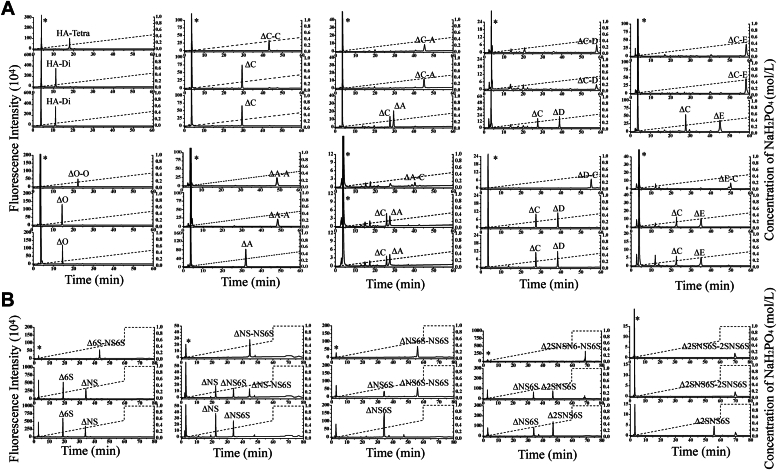
**Sulfation pattern preference of GAGase.***A*, digestion of HA–CS tetrasaccharides HA-Tetra, ΔO-O, ΔC-C, ΔA-A, ΔC-A, ΔA-C, ΔC-D, ΔD-C, ΔC-E, and ΔE-C by GAGase I. Products were labeled with 2-AB and analyzed by anion-exchange HPLC using a YMC-Pack PA-G column. *Top panel*, HA–CS tetrasaccharide; *middle panel*, HA–CS tetrasaccharide digested by GAGase I; *bottom panel*, disaccharide composition of each tetrasaccharide. *B*, digestion of Hep tetrasaccharides Δ6S-NS6S, ΔNS-NS6S, ΔNS6S-NS6S, Δ2SNS6S-NS6S, and Δ2SNS6S-2SNS6S by GAGase I. Products were labeled with 2-AB and detected by anion-exchange HPLC using a YMC-Pack Polyamine II column. *Top panel*, Hep tetrasaccharide; *middle panel*, Hep tetrasaccharide digested by GAGase I; *bottom panel*, disaccharide composition of each tetrasaccharide. The relevant signals are indicated as follows: ∗, the peaks detected around 3 min in the figures are derived from fluorescent labeling reagents; HA-Tetra, HA tetrasaccharide (Δ^4,5^HexUA1-3GlcNAcβ1-4GlcUAβ1-3GlcNAc); HA-Di, HA disaccharide (Δ^4,5^HexUA1-3GlcNAc); ΔO, Δ^4,5^HexUA1-3GalNAc; ΔA, Δ^4,5^HexUA1-3GalNAc(4S), ΔC, Δ^4,5^HexUA1-3GalNAc(6S); ΔD, Δ^4,5^HexUA(2S)1-3GalNAc(6S), ΔE, Δ^4,5^HexUA1-3GalNAc(4S,6S); Δ6S, Δ^4,5^HexUA1-4GlcNAc(6S); ΔNS, Δ^4,5^HexUA1-4GlcNS; ΔNS6S, Δ^4,5^HexUA1-4GlcNS(6S); Δ2SNS6S, Δ^4,5^HexUA(2S)1-4GlcNS(6S). AB, 2-aminobenzamide; CS, chondroitin sulfate; GAG, glycosaminoglycan; GAGase, GAG lyase; HA, hyaluronan.

To investigate the preference of GAGases for the structures of substrates in more detail, various structure-defined GAG tetrasaccharides were prepared as substrates for GAGase I (Table S3). As we speculated, GAGase I could completely degrade HA tetrasaccharide and CS tetrasaccharides ΔO-O and ΔC-C rather than ΔA-A into disaccharides (Fig. 6*A*). However, this enzyme could not cleave ΔC-A, ΔC-D, and ΔC-E but could cleave ΔA-C, ΔD-C, and ΔE-C (Fig. 6*A*), suggesting that the resistant structures A, D, and E units located at the reducing end of the C unit will inhibit the action of GAGases. Furthermore, once ΔC-A or ΔC-E was desulfated with 4-*O*-endosulfatase (22), both could be degraded to generate one ΔO and one ΔC or two ΔC (Fig. S5, *A* and *B*), confirming that the 4-*O*-sulfation of GalNAc was one inhibitory factor for these enzymes. In contrast, even when ΔC-D was desulfated with 6-*O*-exosulfatase (23), the resultant Δ^4,5^HexUA1-3GalNAc(6S)β1-4GlcUA(2S)β1-3GalNAc (ΔC-U) could not be cleaved by GAGase I (Fig. S5*C*), indicating that the 2-*O*-sulfation of GlcUA was another factor inhibiting GAGases. Furthermore, by using sequence-defined Hep/HS tetrasaccharides as substrates (18), we found that GAGase I could digest Δ^4,5^HexUA1-4GlcNAc(6S) 1-4HexUA1-4GlcNS(6S) (Δ6S-NS6S), Δ^4,5^HexUA1-4GlcNS1-4HexUA1-4GlcNS(6S) (ΔNS-NS6S), Δ^4,5^HexUA1-4GlcNS(6S)1-4HexUA1-4GlcNS(6S) (ΔNS6S-NS6S), and Δ^4,5^HexUA2S1-4GlcNS(6S)1-4HexUA1-4GlcNS(6S) (Δ2SNS6S-NS6S) but not Δ^4,5^HexUA(2S)1-4GlcNS(6S)1-4HexUA(2S)1-4GlcNS(6S) (Δ2SNS6S-2SNS6S) (Fig. 6*B*), further confirming that GAGase I can act on the α1-4 bond between nonsulfated HexUA and 6-*O*-sulfated GlcNAc–GlcNS residues in HS–Hep but cannot cleave this bond once the HexUA residue is 2-*O*-sulfated similar to the case of CS. Notably, ΔNS-NS6S, ΔNS6S-NS6S, and Δ2SNS6S-NS6S could not be completely digested by GAGase I, which likely occurs because some of the tetrasaccharides in these fractions contain IdoUA that resists action by GAGase I, as demonstrated later.

### Epimerization preference of GAGases

**Figure 7:**
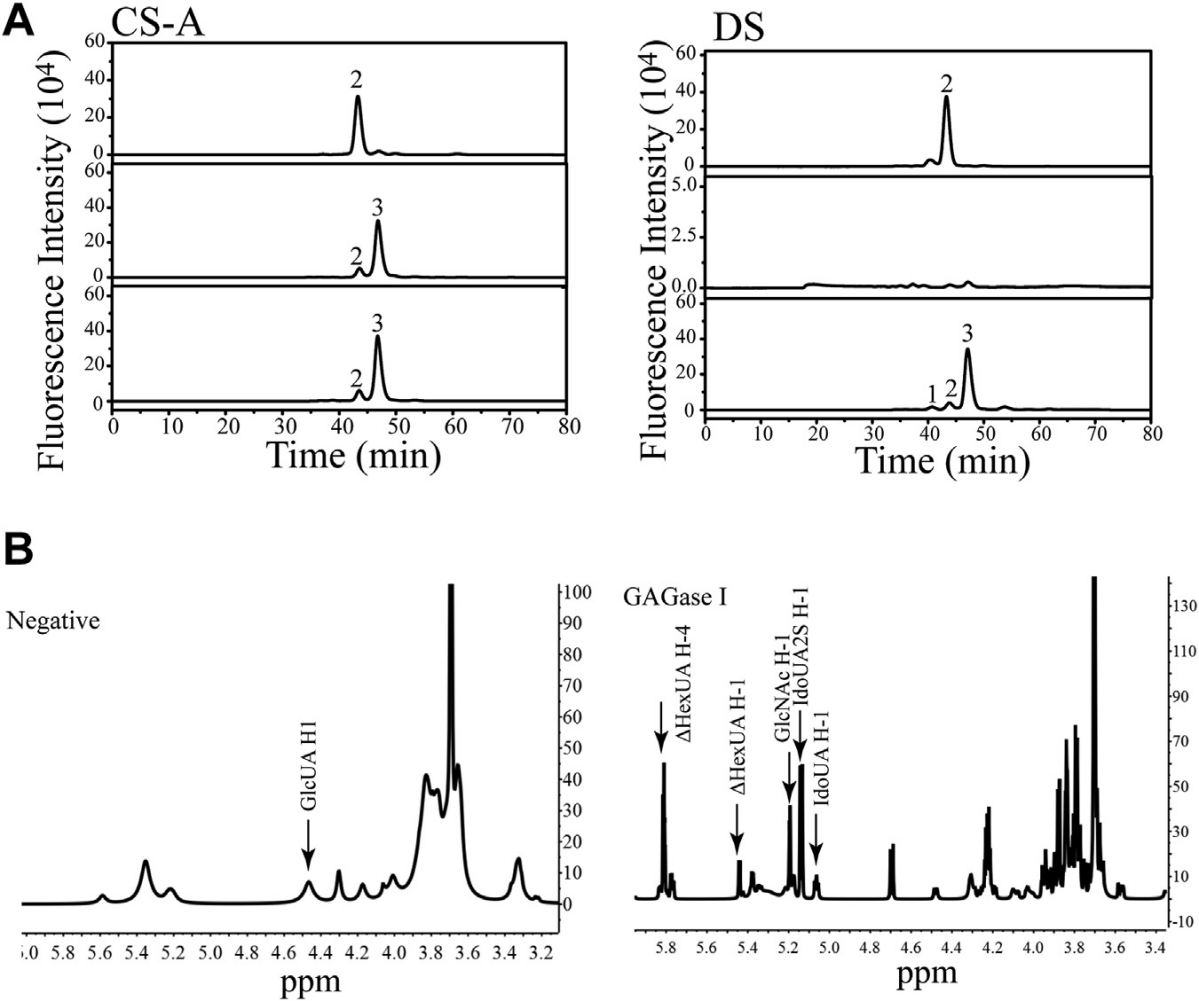
**Epimerization preference of GAGase I for GAGs.***A*, digestion of 4-*O*-desulfated CS-A and DS by GAGase I. Products were labeled with 2-AB and detected by gel filtration HPLC using a Superdex Peptide 10/300 GL column. *Top panel*, CSase ABC treated CS-A/DS; *middle panel*, the GAGase I treated 4-*O*-desulfated CS-A/DS; *bottom panel*, CSase ABC treated 4-*O*-desulfated CS-A/DS. 1, disulfated disaccharide fraction; *2*, monosulfate disaccharide fraction; 3, nonsulfate disaccharide fraction. *B*, the ^1^H NMR analysis of products from HS treated with GAGase I. HS was exhaustively degraded without or with GAGase I at 30 °C for 12 h. The H-1 signals at 4.47, 5.07, and 5.14 ppm indicate that the internal HexUA residues are GlcUA, IdoUA, and IdoUA with 2-*O*-sulfation, respectively. 2-AB, 2-aminobenzamide; CS, chondroitin sulfate; DS, dermatan sulfate; GAG, glycosaminoglycan; GAGase, GAG lyase; HS, heparan sulfate.

Based on the results obtained from polysaccharide degradation (Fig. 3), GAGases cannot easily degrade IdoUA-rich DS and Hep, suggesting that these enzymes are sensitive to the epimerization of GlcUA in GAGs. For further demonstration, GlcUA-containing CS-A and IdoUA-containing DS were first treated with 4-*O*-endosulfatase (22) to exclude the resistance of 4-*O*-sulfation to enzymatic catalysis and then digested by GAGase I. The results showed that GAGase I-like CSase ABC could completely digest 4-*O*-desulfated CS-A into disaccharides but could not act on 4-*O*-desulfated DS (Fig. 7*A*), indicating that GAGase I cannot degrade DS because of the epimerization of GlcUA to IdoUA except for 4-*O*-sulfation. Moreover, the type of HexUA in the final product of HS treated by GAGase I was analyzed with ^1^H NMR spectra. Compared to the untreated HS rich in GlcUA residues, GAGase I-treated HS lost the 4.47 ppm signal corresponding to H1 of GlcUA residues but maintained the 5.07 and 5.14 ppm signals to the H1 of internal IdoUA and IdoUA2S, respectively (Fig. 7*B*), demonstrating that the IdoUA residue in Hep/HS is also a resistance factor for GAGases.

**Figure 8:**
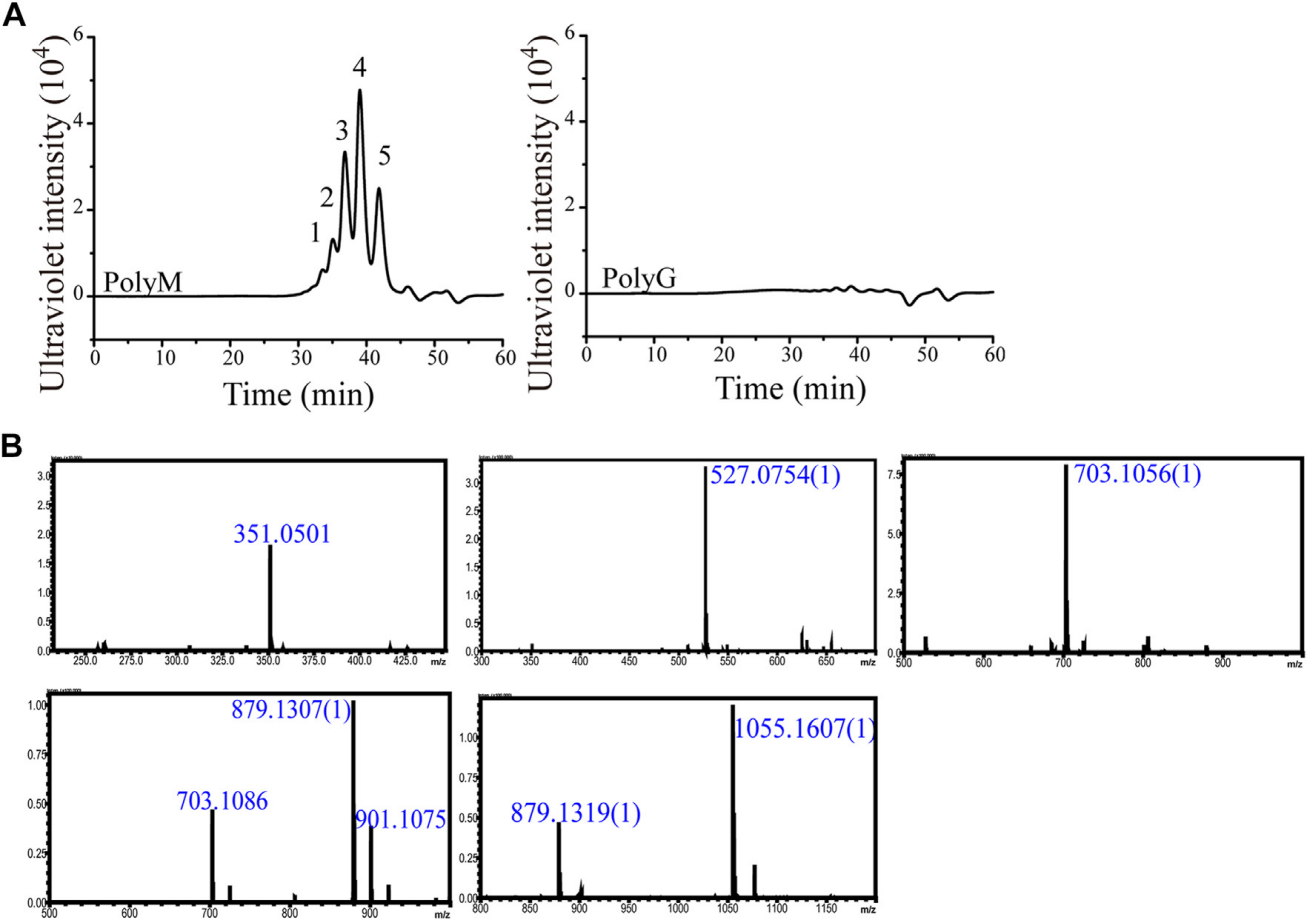
**Epimerization preference of GAGase III for alginate.***A*, digestion of polyM and polyG by GAGase III. PolyM and polyG (30 μg) were individually digested with GAGase III (6 μg) at 30 °C and analyzed by gel filtration chromatography HPLC. Fraction 1 to 5 indicate hexasaccharide to disaccharide. *B*, analysis of the oligosaccharides from the digestion of polyM by GAGase III. The di- to hexasaccharides from the digestion of polyM by GAGase III were collected by gel filtration chromatography, and each fraction was analyzed using liquid chromatography–ion trap time-of-flight hybrid mass spectrometry. The 351.05, 527.07, 703.10, 879.13, and 1055.16 *m*/*z* indicated the unsaturated alginate di- to hexasaccharides. GAG, glycosaminoglycan; GAGase, GAG lyase.

In addition, GAGase III could efficiently degrade the polyM that mainly contained M residues through β1–4 glucosidic bonds but not the polyG that mainly contained G residues through α1–4 glucosidic bonds (Fig. 8*A*). Five well-separated low–molecular-weight oligosaccharides were collected from the digest of the polyM block of alginate by GAGase III, and their molecular ion peaks detected by liquid chromatography–ion trap time-of-flight hybrid mass spectrometry at 351.05, 527.07, 703.10, 879.13, and 1055.16 *m*/*z* could be assigned to unsaturated alginate di- to hexasaccharides, confirming that this enzyme exhibits M block–specific alginate lyase activity (Fig. 8*B*). Therefore, alginate-degrading GAGases are also sensitive to the epimerization of M to G in alginate. Taken together, these results suggest that GAGases are specific to the configuration of HexUA and can act on d-GlcUA/M but not l-IdoUA/G residues in GAG–alginate.

## Discussion

GAG lyases not only play crucial roles in microbial degradation and utilization of GAGs but are also essential tools for structural and functional analysis of GAGs. Currently, a variety of GAG lyases have been identified and classified into 13 PL families in the CAZy database based on their sequences, especially the homology of the catalytic domains (8). In terms of substrate type, these GAG lyases can be divided into three main groups: HA-specific lyases such as HYAL from *Streptomyces hyalurolyticus* (9) or *Streptococcus dysgalactiae* (10), CS/DS lyases CSase AC I and B from *Flavobacterium heparinum* (11) and CSase ABC I and II from *Proteus vulgaris* (12), and HS–Hep lyases such as heparinase I, II, and III from *F. heparinum* (14, 15, 16). Notably, although most of the CS–DS lyases (except DS-specific CSase B) exhibit HA-degrading activity, no GAG lyase has been reported to act on all three types of HexUA-containing GAGs. Undoubtedly, the discovery of GAGases with HA, CS, HS, and even alginate-degrading activities in this study will expand our understanding of the type diversity and broad substrate spectrum of GAG lyases.

Based on sequence analysis, GAGases belong to the recently established PL35 family, in which only one member was reported in a large-scale screening of novel carbohydrate-active enzymes through the rational exploration of the protein sequence space (8). In the GenBank database, most hypothetical proteins belonging to the PL35 family are annotated as “heparinase,” “heparinase II/III-like protein,” or “DUF4962 domain–containing protein,” whereas the only reported member in the PL35 family has been identified as “endo-chondroitin lyase” (17). Phylogenetic analysis showed that the “endo-chondroitin lyase” clusters with GAGases, but it is more distantly related to GAGases, which may explain why it is different from GAGases in substrate selectivity. On the other hand, the capacity of this enzyme to degrade HA and HS might be overlooked because the activity against these substrates is too low for detection by conventional methods, such as the reducing sugar analysis used in a previous study (17). The GAGases reported in this study share very low sequence similarity (<22%) with all identified GAG lyases in other PL families and have a broader substrate spectrum than all these identified GAG lyases, suggesting the discovery of a new class of GAG lyases.

Based on substrate selectivity of various identified GAG lyases, these enzymes should be more sensitive to the type of glycosidic bonds than to the composition of the monosaccharide residues in the chain of GAGs. For example, HA and CS with different monosaccharide compositions but the same type of glycosidic bonds between residues can be degraded simultaneously by many identified HA/CS lyases, whereas enzymes that can degrade HA and HS with the same monosaccharide composition but different glycosidic bonds are rarely reported. In contrast, GAGases exhibit higher tolerance for substrate structure to the stereoisomeric form of glycosidic bonds and can cleave both α- and β-1,4 bonds between basic disaccharide units in the chains of HA, CS, and HS. However, detailed analysis by using various substrates with specific structures revealed that GAGases show strict selectivity for the sulfation pattern and epimerization of monosaccharides in substrates. GAGases prefer to degrade non/6-*O*-sulfated domains in HA/CS/HS but fail to function once the GalNAc residues are 4-*O*-sulfated in CS or the HexUA residues are 2-*O*-sulfated in both CS and HS. Furthermore, GAGases are highly sensitive to the C-5 epimerization of d-GlcUA residues in GAGs and cannot efficiently digest l-IdoUA-enriched DS and Hep, similar to the cases of their structurally homologous CSase AC and heparinase III (13, 15).

More interestingly, GAGase III exhibit remarkable activity against alginate with a completely different structure from GAGs and can only cleave the polyM domain consisting of non-C-5 epimerized d-M residues. To the best of our knowledge, this is the first time that GAG lyases have been found to exhibit alginate-degrading activity. The stronger alginate-degrading activities of GAGase III than other GAGases from gut and soil should result from their origin in the aquatic environment, which is rich in alginate from algae.

Like GAGs, alginate is also a linear polyanionic polysaccharide that contains HexUA residues. Researchers have long been puzzled by why alginate lyases have the “Hepar_II_III superfamily” module but cannot degrade Hep–HS. From an evolutionary perspective, GAG lyases may have originated from alginate lyases through adaptation to the evolution of substrate polysaccharides (24). In this study, a C-terminal “Hepar_II_III superfamily” module was also found in GAGases, suggesting that it is a conserved functional domain during the divergent evolution from alginate lyase to heparinase. Compared with various typical GAG lyases, GAGases exhibit much weaker substrate specificity and thus can degrade three types of HexUA-containing GAGs, even alginate, but only some nonsulfated/hyposulfated and nonepimerized primary structures, These may be functional features of the transition from alginate lyase to GAG lyase. As far as why these GAGases have such broad substrate spectrum, a crystal structure and catalysis mechanism study of GAGases has been achieved in another study.

In conclusion, the discovery of GAGases with an ultrabroad substrate spectrum not only changes our understanding of the substrate limitations of GAG lyases and further enriches the types of GAG lyases but also provides potential candidates for studying the evolution of GAG lyases.

## Experimental procedures

### Materials

HA from *Streptococcus equi* (average molecular weight [MW]: ∼1650 kDa), CS-A from bovine trachea (average MW: ∼30 kDa), CS-C from shark cartilage (average MW: ∼55 kDa), alginate sodium from brown algae (average MW: ∼270 kDa), 2-aminobenzamide (2-AB), sodium cyanoborohydride, CSase ABC from *P. vulgaris*, and heparinase I/II/III (hepase I/II/III) from *F. heparinum* were purchased from Sigma‒Aldrich. CS-D was extracted from shark fin (average MW: 55 kDa) (25). CS-E (average MW: ∼700 kDa) was extracted from squid cartilage (26). DS (average MW: ∼35 kDa), Hep (average MW: ∼15 kDa), and HS (average MW: ∼50 kDa) from porcine intestinal mucosa were provided by Tiandong Pharma. PolyM/G from alginate was prepared following the methodology described in a previous study (27). The unsaturated HA tetrasaccharide and the unsaturated CS tetrasaccharides (ΔA-A, ΔC-C, ΔD-C, and ΔC-A) were separated from the final products of enCSase toward corresponding polysaccharide substrates (28); the nonsulfated tetrasaccharide (ΔO-O) was prepared by treating ΔA-A with GalNAc-4-*O*-endosulfatase (22); ΔC-D, ΔC-E, and ΔA-C were prepared from partially digesting CS-D and CS-E by HCLase (29); ΔE-C and ΔE-A were prepared from the final digests of CS-E by HCLase Er (30); Hep tetrasaccharides, including Δ6S-NS6S, ΔNS-NS6S, ΔNS6S-NS6S, Δ2SNS6S-NS6S, and Δ2SNS6S-2SNS6S, were prepared by partially digesting Hep with heparinase II (17) (Table S3). All these tetrasaccharides were separated by gel filtration chromatography and then subfractionated by anion-exchange chromatography using a YMC-Pack PA-G column or a YMC-Pack Polyamine II column. GAGs from normal 293T cells and 4T1 cancer cells were extracted as reported previously (31, 32).

The putative genes of GAGases were synthetized by Genewiz Biotech Co, Ltd. The putative gene of GAGase VII was amplified from the genome of enteric bacterium *Bacteroides intestinalis.* DSM 17393 was purchased from German Collection of Microorganisms and Cell Cultures (DSMZ) (Table S4). All genes were constructed into pET-30a expression plasmid with kanamycin resistance and fused with a His_6_ tag at its 3′-terminal region. The PrimeSTAR HS DNA polymerase, restriction endonucleases, and T4 ligase were purchased from Takara, Inc. The Genome Extraction Kit was purchased from Tiangen Biotech, Co, Ltd. The competent *E. coli* BL21 (DE3) cells and Fast Mutagenesis Kit V2 were purchased from Vazyme Biotech Co, Ltd.

### Sequence analyses of PL35 family GAGases

The gene and protein sequences of PL35 family proteins in this study were downloaded from the National Center for Biotechnology Information database (https://www.ncbi.nlm.nih.gov/). Codon optimization was performed by Codon OptimWiz developed by Genewiz Biotech Co, Ltd. The sequence similarity and functional module analyses of the protein sequences were performed using the Protein BLAST online (https://blast.ncbi.nlm.nih.gov/Blast.cgi). Secretion signal peptide was analyzed using the SignalP 5.0 server (https://services.healthtech.dtu.dk/service.php?SignalP-5.0) (33). The molecular mass of the protein was estimated using Compute pI/MW tool on the ExPASy server of the Swiss Institute of Bioinformatics (https://www.expasy.org/) (34). Multiple sequence alignment and phylogenetic analysis were performed using BioEdit, version 7.0.5.3 (https://thalljiscience.github.io/) and MEGA, version 7.0 (35). The classification of these enzymes was confirmed based on the database of carbohydrate-active enzymes (CAZy) (http://www.cazy.org/) (8).

### Heterologous expression and purification of GAGases

The synthesized putative PL35 family genes (*GAGase I*-*GAGase VI* and *GAGase VIII*) were inserted into expression plasmid pET-30a (+). The recombinant *GAGase VII* was constructed starting with PCR from the genome of *B. intestinalis* DSM 17393 (Table S4). Plasmid pET-30a-GAGases were transformed into *E. coli* BL21 (DE3) cells, and their fragment integrity was confirmed by DNA sequencing. To express GAGases, *E. coli* cells harboring the expression vector pET-30a-GAGases were expanded in LB broth at 37 °C. The putative enzymes were induced and expressed at 16 °C for 16 h by supplementing with 5 mM IPTG to a final concentration of 0.05 mM when the cell density reached an absorbance of 0.6 to 0.8 at 600 nm. After further induced cultivation for 16 h, cells were harvested by centrifugation at 8000*g* for 5 min and washed twice with ice-cold buffer A (50 mM Tris–HCl, 150 mM NaCl [pH 8.0]). Then, cells were resuspended and disrupted by sonication (50 repetitions, 4 s) in an ice-cold environment. Cell lysate was separated by centrifugation at 15,000*g* for 30 min at 4 °C. The supernatant containing the soluble putative enzyme was collected and loaded on a nickel affinity column (Nickel-Sepharose 6 Fast Flow resin; GE Healthcare). Then, the column was washed with buffer A containing 10 mM imidazole to remove impurities, and the target protein was finally eluted with buffer A containing 250 mM imidazole. After ultrafiltration using an Amicon Ultra 0.5-ml 10K unit (Millipore) by centrifugation, the buffer was exchanged to buffer A for biochemical characteristic assays. The expression and purity of PL35 GAGases were analyzed using 13.2% SDS-PAGE followed by Coomassie Brilliant Blue R-250 stain. The concentration of each protein was determined using BCA Protein Assay Kit (CWBIO).

### Activity of GAGases toward various GAGs and alginate

GAGs (*e.g.*, HA, CS-A, CS-C, CS-D, CS-E, DS, Hep, and HS) and alginate (*e.g.*, alginate, polyM, and polyG) (30 μg) were used as substrates for GAGases. Each reaction (30 μl) containing the corresponding substrate and purified GAGase (6 μg) in 50 mM Tris–HCl (pH 7.0) buffer was incubated at 30 °C overnight. After inactivation by boiling for 10 min, the products were centrifuged at 15,000*g* for 10 min. The supernatants were collected and analyzed by gel filtration HPLC using a Superdex Peptide 10/300 GL column with 0.20 M NH_4_HCO_3_ as the mobile phase at a flow rate of 0.4 ml/min. The eluates were monitored at 232 nm using a UV detector and analyzed online using the LCsolution, version 1.25, software. Each experiment was set up with an enzyme inactivated by heating at 100 °C as a negative control to exclude the possible effect from the nonspecific degradation of the substrate caused by reaction conditions. The size-defined oligosaccharide fractions of each substrate were individually collected and desalted by repeating freeze-drying for the following disaccharide composition and mass spectrometry analysis. In addition, GAGs (3 μg) extracted from 4T1 or 293T cells were digested with GAGase I at 30 °C overnight, and the digest was labeled with 2-AB and analyzed by gel filtration HPLC using a Superdex Peptide 10/300 GL column as described previously (36, 37).

The disaccharide compositions of HA/CS and HS oligosaccharides were analyzed by complete digestion with CSase ABC and with heparinase (I, II, and III), respectively, followed by 2-AB labeling and anion-exchange HPLC as described previously (18, 22). Briefly, each oligosaccharide fraction was completely digested with CSase ABC or with heparinases, and the digest was labeled with 2-AB (36, 37). After the free 2-AB was removed by extraction with chloroform, the sample was analyzed by anion-exchange HPLC on a YMC-Pack PA-G column or a YMC-Pack Polyamine II column eluted with a linear gradient from 16 to 460 mM or 16 to 550 mM NaH_2_PO_4_ over a 60 min period at a flow rate of 1 ml/min. Elutes were monitored using a fluorescence detector with excitation and emission wavelengths of 330 and 420 nm, respectively. The disaccharide composition was determined by calculating the ratio of the peak area of each disaccharide to the peak area of all disaccharides.

The disaccharide fractions from the digestion of GAGs or the di- to hexasaccharide fractions from the digestion of polyM were individually analyzed by electrospray ionization mass spectrometry on a liquid chromatogram-ion trap-time of flight mass spectrometer with negative-ionization modes with a nebulizing gas flow of 1.5 L/min and a detector voltage at 1.75 kV. The mass acquisition range was set at 100 to 1200.

### Biochemical characterization of the PL35 family GAGases

Using HA as the substrate, to determine the optimal temperature, the activities of GAGases (7.5 μl) were measured with 150 μg HA (1 mg/ml) in 50 mM Tris–HCl (pH 7.0) at temperature from 0 to 70 °C for 1 h in a total volume of 150 μl. The optimal pH of GAGases was determined with various buffer with different pH values, including 50 mM NaAc–HAc buffer (pH 5.0–6.0), 50 mM NaH_2_PO_4_–Na_2_HPO_4_ buffer (pH 6.0–8.0), and 50 mM Tris–HCl buffer (pH 7.0–10.0) at their optimal temperature for 1 h. The effects of metal ions/chelating reagent (5 mM) on the activity of each enzyme were investigated at their optimal temperature and pH. The thermostability of GAGase I or GAGase III was determined in 50 mM Tris–HCl buffer (pH 7.0) by preincubating these enzymes at a temperature from 0 to 70 °C for 0 to 24 h, and the residual activities were determined by incubating the mixtures at 40 °C for 1 h. All reactions were carried out triplicate, and the activities under each reaction condition were estimated by measuring the absorbance at 232 nm after denatured by boiling for 10 min.

In addition, the reaction rates of GAGase I–V against HA, CS-C, HS, and alginate were measured by treating these substrates (the final concentrations of 0–4 mg/ml) with enzymes (2.5–30 μg) under the optimal conditions for 1 h. Enzymes were denatured by boiling. The products were analyzed by measuring the absorbance at 232 nm. The kinetic parameters were calculated based on the Michaelis–Menten equation fitting with OriginPro, version 2022 (https://www.originlab.com/). The fitting curves are presented in the “Supporting information” section (Fig. S6).

### Specific activity assay of GAGases

The specific activities of GAGases were determined by using a series of GAGs (HA, CS-A, CS-C, and HS) and alginate as substrates under their optimal conditions. Each reaction, containing 1 mg/ml substrates and 1 μg/μl purified GAGases in a total volume of 1 ml, was incubated in the optimal conditions of the corresponding enzyme. Aliquots of 150 μl were withdrawn, boiled for 10 min, and then cooled in ice-cold water for 10 min at 0, 0.5, 1, 2, and 5 min. The supernatants were collected after being centrifuged at 15,000*g* for 10 min and analyzed by detecting absorbance at 232 nm using UV spectrophotometer. All reactions were performed in triplicate, and the activities toward different substrates were calculated based on the changes of the 232 nm absorbance. One unit was defined as the amount of enzyme that produced 1 μmol of unsaturated carbon bonds per min.

### Action pattern of GAGases

The action pattern of GAGase I was confirmed by time-course experiments, and the digests of HA (1 mg, 1 mg/ml) by each GAGase (200 μg, 2 mg/ml) were traced at 40 °C. Aliquots of the products (30 μg) were removed every hour to analyze product-generating patterns. Samples were analyzed by gel filtration chromatography on a Superdex Peptide 10/300 GL column monitored at 232 nm as described previously.

### Sulfation pattern preference of GAGase I

To determine the preference of GAGase I for the sulfation pattern of GAGs, a series of sequence-defined HA/CS tetrasaccharides, including HA-Tetra, ΔO-O, ΔA-A, ΔC-C, ΔC-A, ΔA-C, ΔD-C, ΔC-D, ΔE-C, ΔC-E, and Hep tetrasaccharides, including Δ6S-NS6S, ΔNS-NS6S, ΔNS6S-NS6S, Δ2SNS6S-NS6S, and Δ2SNS6S-2SNS6S, were treated with GAGase I (2 μg) at 30 °C overnight (Table S3). The reactants were boiled for 10 min, labeled with 2-AB, and individually analyzed by anion-exchange HPLC on a YMC-Pack PA-G column or a YMC-Pack Polyamine II column monitored with a fluorescence detector as described previously. To confirm the inhibitory effect of 4-*O*-sulfation on enzymatic activity, the resistant tetrasaccharides ΔC-A and ΔC-E were treated with 4-*O*-endosulfatase (2 μg) to remove the 4-*O*-sulfate groups (22), and the resultants were further treated with GAGase I (2 μg) and analyzed by HPLC as aforementioned. To determine whether 2-*O*-sulfation or high sulfation inhibits the action of GAGase I, ΔC-D was treated with GalNAc-6-*O*-exosulfatase to produce Δ^4,5^HexUA1-3GalNAc(6S)β1-4GlcUA(2S)β1-3GalNAc (ΔC-U) (23) and incubated with GAGase I (2 μg) overnight. The final reactant was labeled by 2-AB and analyzed by anion-exchange HPLC as aforementioned.

### Epimerization preference of GAGase I

To investigate whether GAGase I acts only on GlcUA but not its epimer IdoUA, CS-A and DS were 4-*O*-desulfated by pretreatment with a 4-*O*-endosulfatase (22), which first eliminates the inhibitory effect of 4-*O*-sulfate groups on the enzyme. After denaturation by boiling, the reactants were further treated with GAGase I (2 μg) at 30 °C overnight, and the final products were labeled with 2-AB and analyzed by gel filtration HPLC on a Superdex Peptide 10/300 GL column eluted with 0.20 M NH_4_HCO_3_ at a flow rate of 0.4 ml/min and monitored using a fluorescence detector with excitation and emission wavelengths of 330 and 420 nm. In addition, HS (50 mg) was exhaustively treated with or without GAGase I (5 mg) and purified by deproteinization with 5% trichloroacetic acid, precipitation with two volumes of ethanol, and desalting with a PD-10 column (22). The purified HS samples were individually suspended in 0.5 ml of 99.9% D_2_O and analyzed with ^1^H-NMR. The ^1^H-NMR spectra (500 MHz) were recorded on an Agilent DD2 600 MHz spectrometer at 298 K. To determine the selectivity of GAGases toward M- and G-containing domains of alginate, polyM or polyG (30 μg) was treated with GAGase III (6 μg) at 30 °C overnight, and the resultants were analyzed by gel filtration HPLC as described previously.

### Preparation of oligosaccharides from the final products of GAGase I

To isolate the final products of GAGase I, GAGs (100 mg) including CS-A, CS-C, CS-E, and HS were exhaustively digested by GAGase I (8 U) at 30 °C for 24 h. Reactions were inactivated by boiling for 10 min, and the supernatants were individually loaded on a Superdex Peptide 10/300 GL column eluted with 0.2 M NH_4_HCO_3_ at a 0.4 ml/min flow rate. The eluted oligosaccharide fractions were individually collected based on online monitoring at 232 nm, desalted by three times’ repeating freeze-drying, and finally dissolved in deionized water.

### Sequencing of the tetrasaccharides in the final products of GAGase I

The CS tetrasaccharide fraction isolated from the final products of GAGase I was further subfractionated by anion-exchange HPLC on a YMC-Pack PA-G column eluted by a linear gradient from 16 to 460 mM NaH_2_PO_4_ over 60 min at a flow rate of 1.0 ml/min. The major peaks were collected and desalted with a Superdex Peptide 10/300 GL column monitored at 232 nm. The obtained tetrasaccharides were sequenced by an enzymatic method developed previously. Briefly, the disaccharide composition of each resistant tetrasaccharide was determined by digestion with CSase ABC followed by labeling with 2-AB and analyzing by anion-exchange HPLC on a YMC-Pack PA-G column eluted with a linear gradient from 16 to 460 mM NaH_2_PO_4_ over a 60 min period and monitored using a fluorescence detector; the disaccharide at the reducing end of each tetrasaccharide was identified by degrading the 2-AB-labeled tetrasaccharide with HCDLase followed by HPLC analysis as aforementioned; the sequence of each resistant tetrasaccharide was deduced by combining the analysis results of the disaccharide composition and the disaccharide at the reducing end.

## Data availability

All data supporting the findings of this study are available within the article (and supporting information files). All relevant data generated during this study or analyzed in this published article (and supporting information files) are available from the corresponding author on reasonable request. The sequences of GAGase I (GenBank: ADB38475.1), GAGase II (GenBank: SOD82962.1), GAGase III (GenBank: MBC7922758.1), GAGase IV (GenBank: MPR36080.1), GAGase V (GenBank: HCY41582.1), GAGase VI (GenBank: EKX96148.1), GAGase VII (GenBank: EDV05210.1), and GAGase VIII (GenBank: AVM53611.1) already exist in the National Center for Biotechnology Information database.

## Supporting information

This article contains supporting information.

## Conflict of interest

The authors declare that they have no conflicts of interest with the contents of this article.
