# Supplementary material for: Discovery of a class of glycosaminoglycan lyases with ultrabroad substrate spectrum and their substrate structure preferences
Source: J Biol Chem. 2024 Jun 12;300(7):107466. doi: 10.1016/j.jbc.2024.107466 (PMC11262172; doi:10.1016/j.jbc.2024.107466)
Supplement: Supporting information [file mmc1.docx]

Supporting Information for

**Discovery of a class of glycosaminoglycan lyases with** **ultrabroad substrate spectrum and their substrate structure preferences**

*Lin Wei^1^, Ruyi Zou^1^, Min Du^1^, Qingdong Zhang^2^,* *Danrong Lu^2^,* *Yingying Xu^1^, Xiangyu Xu^1^,* *Wenshuang Wang^1^,* *Yu-Zhong Zhang^3,4,5^** *,* *Fuchuan Li^1,5^**

^1^National Glycoengineering Research Center and Shandong Key Laboratory of Carbohydrate Chemistry and Glycobiology, Shandong University, Qingdao, China.

^2^School of Life Science and Technology, Weifang Medical University, Weifang, China.

^3^MOE Key Laboratory of Evolution and Marine Biodiversity, Frontiers Science Center for Deep Ocean Multispheres and Earth System & College of Marine Life Sciences, Ocean University of China, Qingdao, China.

^4^Marine Biotechnology Research Center, State Key Laboratory of Microbial Technology, Shandong University, Qingdao, China.

^5^Joint Research Center for Marine Microbial Science and Technology, Shandong University and Ocean University of China, Qingdao, China.

* Corresponding authors E-mail addresses: zhangyz@sdu.edu.cn (Y. Z.); fuchuanli@sdu.edu.cn (F. L.)

Figures


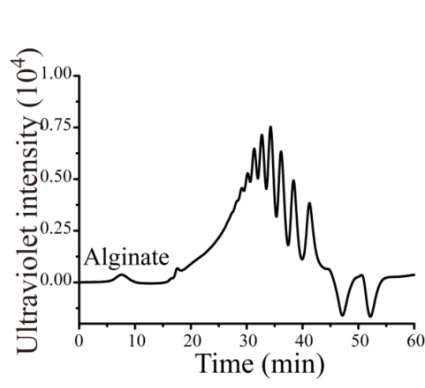


**Figure S1. Digestion of alginate by GAGase III.** Alginate (30 μg) was digested with GAGase III (6 μg) at 30 °C and analysed by gel filtration chromatography HPLC.


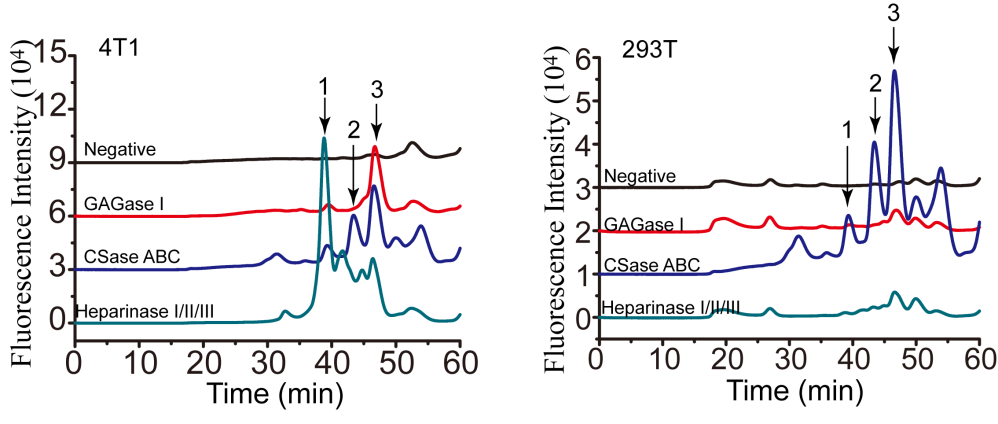


**Figure S2.** **Product analysis of cancer or normal cell-derived GAGs digested with GAGase I.** GAGs extracted from 4T1 or 293T cells were treated with or without GAGase I, CSase ABC and heparinase I/II/III mixture, respectively. Each reactant was labeled with 2-AB and analyzed by gel filtration HPLC using a Superdex^TM^ Peptide 10/300 GL column, and the elutes were detected using a fluorescence detector. The relevant signals are indicated as follows: 1, unsaturated GAG tetrasaccharides; 2, mono-sulfated GAG disaccharides; 3, non-sulfated GAG disaccharides.


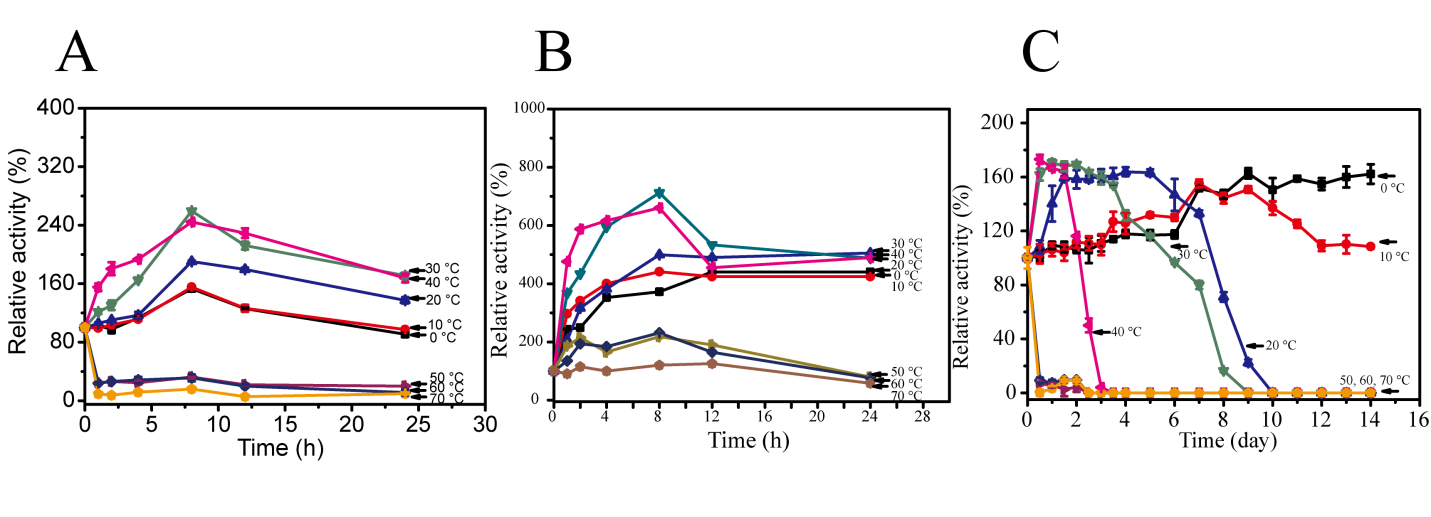


**Figure S3. The thermostability of** **GAGase I and GAGase IV.** The thermostabilities of GAGase I (A, C) and GAGase IV (B) were estimated by incubating this enzyme in 50 mM Tris-HCl buffer (pH 7.0) at various temperatures (0–70 °C) for 24 h (A, B) or 0-14 days (C). The residual activities were measured using HA as substrates (1 mg/ml) in 50 mM Tris-HCl buffer (pH 7.0) at 40 °C for 1 h. The relative activity was calculated by comparing with the activity of the corresponding unpretreated enzyme. *Error bars* represent means of triplicates ± S.D.

**
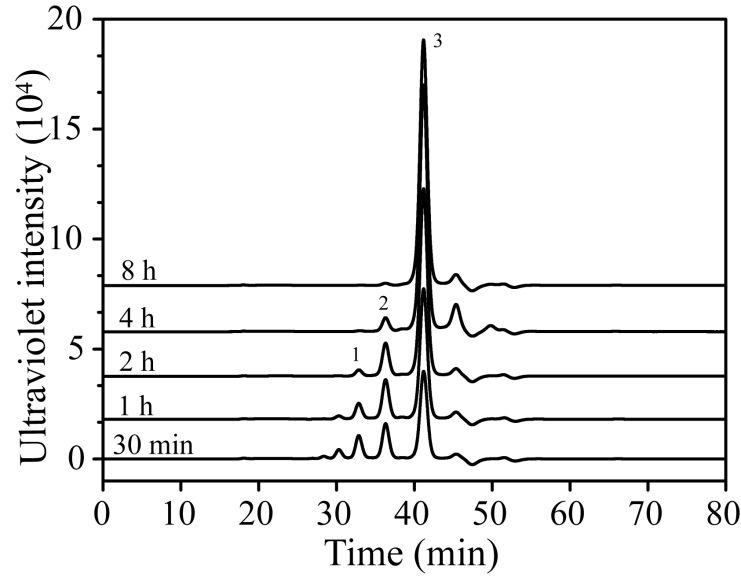
**

**Figure S4. Time-course experiments of degradation of HA by GAGase I.** HA (1 mg/ml) was digested by GAGase I in 50 mM Tris-HCl (pH 7.0) buffer at 40 °C, and 30-μl aliquots were taken at different time points for gel filtration chromatography analysis. The elution positions of the following oligosaccharides are indicated: 1, HA hexasaccharide; 2, HA tetrasaccharide; 3, HA disaccharide.

**
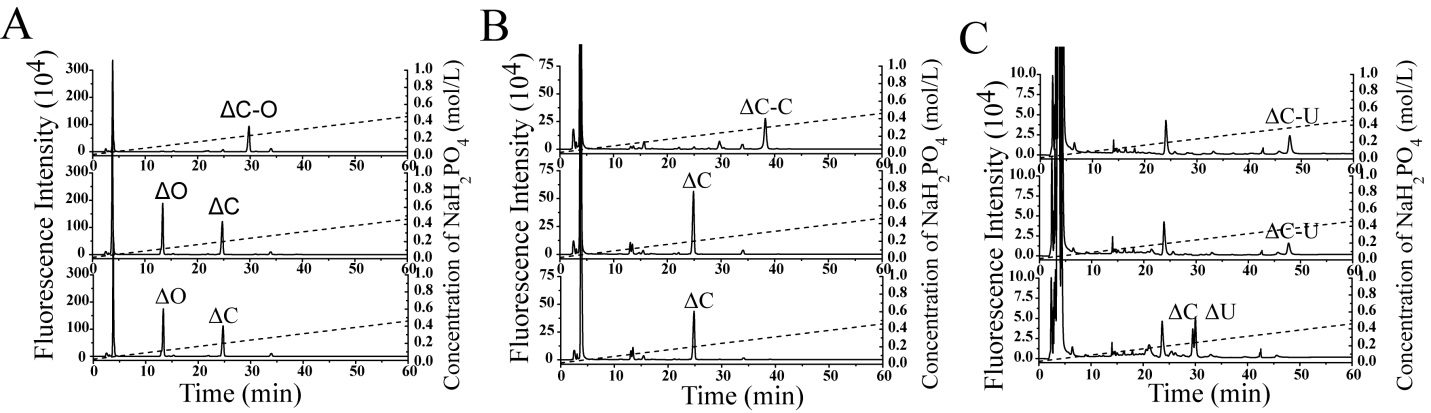
**

**Figure S5. Digestion of desulfated CS tetrasaccharides by GAGase I.** The 4-*O*-sulfate groups of ΔC-A (A), ΔC-E (B) and the reducing end 6-*O*-sulfate group of ΔC-D (C) were removed with 4-*O*-endosulfatase and 6-*O*-exosulfatase, respectively, and the desulfated tetrasaccharides ΔC-O, ΔC-C and ΔC-U were further treated with GAGase I. *Top panel*, the CS tetrasaccharide digested with sulfatase; *middle panel*, the desulfated tetrasaccharide digested with GAGase I; *bottom panel*, the desulfated tetrasaccharide digested with CSase ABC. The relevant signals are indicated as follows: ΔO, Δ^4,5^HexUA1–3GalNAc; ΔC, Δ^4,5^HexUA1–3GalNAc(6S); ΔU, Δ^4,5^HexUA(2S)1–3GalNAc.


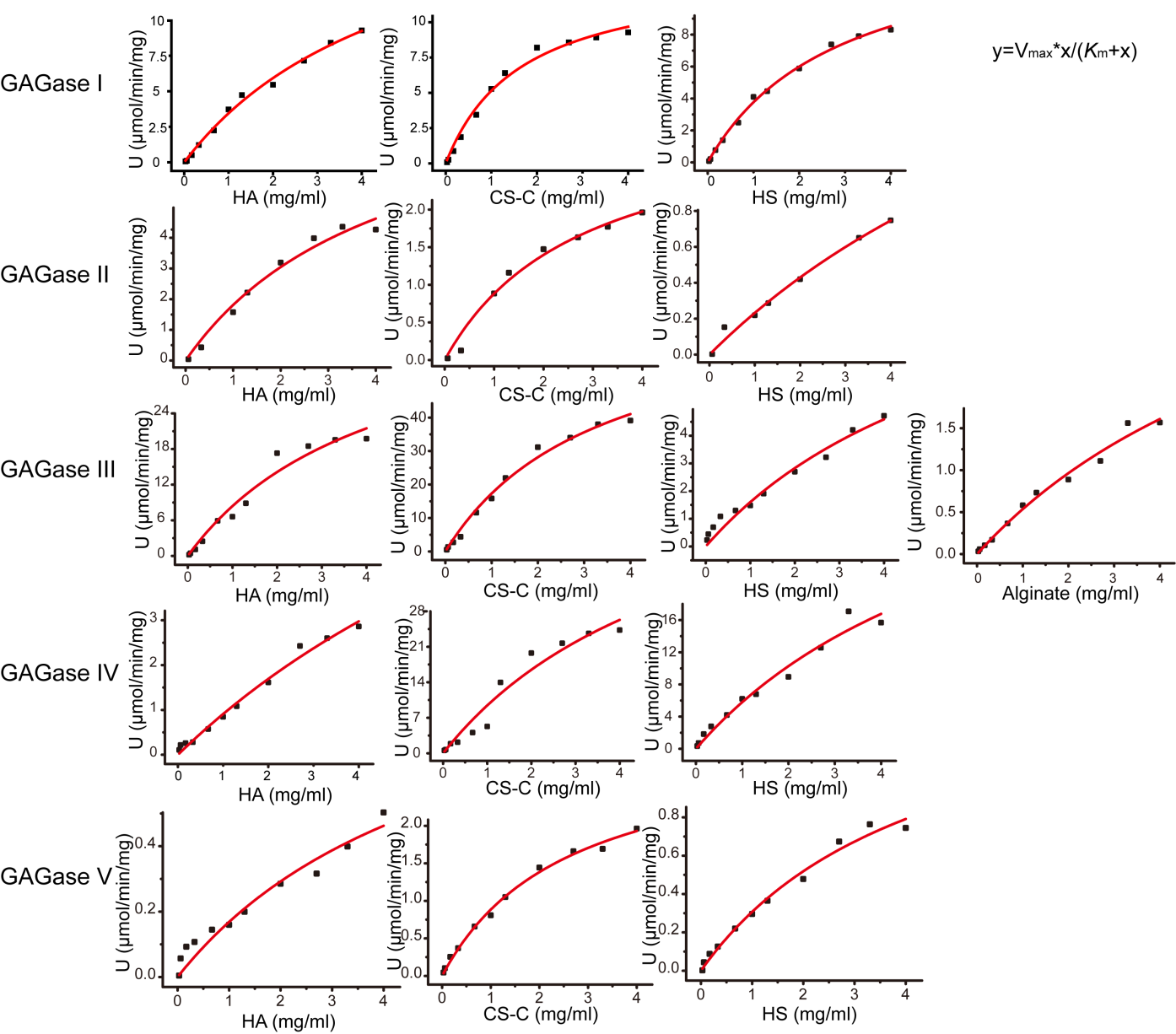
**Figure S6.** **Apparent kinetic analysis of GAGase I-V toward HA, CS-C, HS and alginate.** Reactions were performed using various concentration of HA, CS-C and HS (0-4 mg/ml) with GAGase I-IV (2.5-12 μg) in 50 mM Tris-HCl buffer (pH 7.0) at 40 °C. The Michaelis-Menten equation was fitted using originpro version 2022.

**Tables**

**Table S1. Disaccharide compositions of CS/DS and Hep/HS used as substrates.**

| CS/DS (mol%) | 0S | 4S | 6S | 2S6S | 2S4S | 4S6S** |
| --- | --- | --- | --- | --- | --- | --- |
| CS-A | 5.5 | 76.0 | 18.5 | N.D. | N.D. | N.D. |
| CS-C | 2.3 | 39.6 | 39.1 | 19.0 | N.D. | N.D. |
| CS-D | 1.7 | 27.9 | 52.3 | 18.1 | N.D. | N.D. |
| CS-E | 5.7 | 39.9 | 18.6 | N.D. | N.D. | 35.8 |
| DS | 1.7 | 89.2 | N.D. | 3.4 | 5.7 | N.D. |

* N.D. means not detected.

** 0S, HexUA1-3GalNAc; 4S, HexUA1-3GalNAc(4S); 6S, HexUA1-3GalNAc(6S); 2S6S, HexUA(2S)1-3GalNAc(6S); 2S4S, HexUA(2S)1-3GalNAc(4S); 4S6S, HexUA1-3GalNAc(4S,6S)

| Hep/HS (mol%) | 0S | 6S | 2S | NS | 2S6S | 6SNS | 2SNS | 2SNS6S** |
| --- | --- | --- | --- | --- | --- | --- | --- | --- |
| Hep | 4.8 | 4.5 | 0.3 | 6.8 | 0.1 | 13.4 | 8.3 | 61.8 |
| HS | 36.3 | 10.2 | 1.3 | 29.5 | 0.6 | 3.5 | 12.7 | 5.9 |

* N.D. means not detected.

** 0S, HexUA1-4GlcNAc; 6S, HexUA1-4GlcNAc(6S); 2S, HexUA(2S)1-4GlcNAc; NS, HexUA1-4GlcNS; 2S6S, HexUA(2S)1-4GlcNS(6S); NS6S, HexUA1-4GlcNS(6S); 2SNS, HexUA(2S)1-4GlcNS; 2SNS6S, HexUA(2S)1-4GlcNS(6S).

**Table S2.** **Disaccharide compositions of oligosaccharide fractions produced from the digestion of GAGs by GAGase I.**

| CS disaccharide unit** | O unit | A unit | C unit | D unit | E unit |
| --- | --- | --- | --- | --- | --- |
|  | (mol%) | | | | |
| CS-A-Polysaccharide | 5.5 | 76.0 | 18.5 | N.D.* | N.D. |
| CS-A-fraction 1 | N.D. | 97.8 | 2.2 | N.D. | N.D. |
| CS-A-fraction 2 | N.D. | 95.2 | 4.8 | N.D. | N.D. |
| CS-A-fraction 3 | N.D. | 91.4 | 8.6 | N.D. | N.D. |
| CS-A-fraction 4 | N.D. | 84.8 | 15.2 | N.D. | N.D. |
| CS-A-fraction 5 | N.D. | 81.6 | 18.4 | N.D. | N.D. |
| CS-A-Octasaccharide | N.D. | 77.6 | 22.4 | N.D. | N.D. |
| CS-A-Hexasaccharide | N.D. | 70.4 | 29.6 | N.D. | N.D. |
| CS-A-Tetrasaccharide | N.D. | 50.6 | 49.4 | N.D. | N.D. |
| CS-A-Disaccharide | 42.9 | 0 | 57.1 | N.D. | N.D. |
| CS-C-Polysaccharide | 2.3 | 39.6 | 39.1 | 19.0 | N.D. |
| CS-C-Fraction 1 | N.D. | 77.7 | 3.6 | 18.7 | N.D. |
| CS-C-Fraction 2 | N.D. | 71.9 | 4.9 | 23.2 | N.D. |
| CS-C-Fraction 3 | N.D. | 66.9 | 7.5 | 25.6 | N.D. |
| CS-C-Fraction 4 | N.D. | 63.8 | 10.3 | 25.9 | N.D. |
| CS-C-Fraction 5 | N.D. | 60.3 | 14.2 | 25.5 | N.D. |
| CS-C-Octasaccharide | N.D. | 55.1 | 19.1 | 25.8 | N.D. |
| CS-C-Hexasaccharide | N.D. | 44.1 | 26.6 | 29.3 | N.D. |
| CS-C-Tetrasaccharide | N.D. | 48.7 | 36.3 | 15.0 | N.D. |
| CS-C-Disaccharide | 5.3 | N.D. | 93.9 | 0.8 | N.D. |
| CS-E-Polysaccharide | 5.7 | 39.9 | 18.6 | N.D. | 35.8 |
| CS-E-Fraction 1 | N.D. | 69.8 | 1.0 | N.D. | 29.2 |
| CS-E-Fraction 2 | N.D. | 60.5 | 2.5 | N.D. | 37.0 |
| CS-E-Fraction 3 | N.D. | 49.5 | 7.0 | N.D. | 43.5 |
| CS-E-Fraction 4 | N.D. | 39.4 | 13.5 | N.D. | 47.1 |
| CS-E-Fraction 5 | N.D. | 33.6 | 15.3 | N.D. | 51.1 |
| CSE-Fraction 6 | N.D. | 28.3 | 21.9 | N.D. | 49.8 |
| CS-E-Octasaccharide | N.D. | 25.0 | 26.5 | N.D. | 48.5 |
| CS-E-Hexasaccharide | N.D. | 31.1 | 29.8 | N.D. | 39.1 |
| CS-E-Tetrasaccharide | N.D. | 25.2 | 43.4 | N.D. | 31.4 |
| CS-E-Disaccharide | 27.2 | 6.8 | 56.1 | N.D. | 9.9 |

*N.D. means not determined.

**O unit, HexUA1-3GalNAc; A unit, HexUA1-3GalNAc(4S); C unit, HexUA1-3GalNAc(6S); D unit, HexUA(2S)1-3GalNAc(6S); E unit, HexUA1-3GalNAc(4S,6S).

| HS disaccharide unit** | 0S | 6S | NS | 6SNS | 2SNS | 2SNS6S** |
| --- | --- | --- | --- | --- | --- | --- |
|  | (mol%) | | | | | |
| HS-Polysaccharide | 36.3 | 10.2 | 29.5 | 3.5 | 12.7 | 5.9 |
| HS-Fraction 1 | N.D. | N.D. | N.D. | 10.1 | 82.3 | 7.6 |
| HS-Fraction 2 | N.D. | 11.0 | 20.9 | 9.5 | 49.6 | 9.0 |
| HS-Fraction 3 | N.D. | 15.3 | 26.8 | 9.3 | 37.4 | 11.2 |
| HS-Fraction 4 | 2.0 | 17.0 | 27.7 | 9.4 | 30.5 | 13.4 |
| HS-Octasaccharide | 25.4 | 18.1 | 24.6 | 5.4 | 15.7 | 10.8 |
| HS-Hexasaccharide | 25.8 | 21.9 | 37.0 | 2.2 | 9.8 | 3.3 |
| HS-Tetrasaccharide | 19.7 | 22.1 | 55.5 | 0.6 | 2.1 | N.D. |
| HS-Disaccharide | 30.4 | 12.7 | 54.7 | 2.2 | N.D. | N.D. |

*N.D. means not determined.

** 0S, HexUA1-4GlcNAc; 6S, HexUA1-4GlcNAc(6S); NS, HexUA1-4GlcNS; NS6S, HexUA1-4GlcNS(6S); 2SNS, HexUA(2S)1-4GlcNS; 2SNS6S, HexUA(2S)1-4GlcNS(6S).

**Table S3. Sequence-defined unsaturated tetrasaccharides used as substrates.**

|  | Abbreviation | Oligosaccharide Sequence |
| --- | --- | --- |
| HA tetrasccharide | HA-Tetra | Δ^4,5^HexUA1-3GlcNAcβ1-4GlcUAβ1-3GlcNAc |
| CS tetrasccharide | ΔO-O | Δ^4,5^HexUA1-3GalNAcβ1-4GlcUAβ1-3GalNAc |
|  | ΔC-C | Δ^4,5^HexUA1-3GalNAc(6S)β1-4GlcUAβ1-3GalNAc(6S) |
|  | ΔA-A | Δ^4,5^HexUA1-3GalNAc(4S)β1-4GlcUAβ1-3GalNAc(4S) |
|  | ΔC-A | Δ^4,5^HexUA1-3GalNAc(6S)β1-4GlcUAβ1-3GalNAc(4S) |
|  | ΔA-C | Δ^4,5^HexUA1-3GalNAc(4S)β1-4GlcUAβ1-3GalNAc(6S) |
|  | ΔC-D | Δ^4,5^HexUA1-3GalNAc(6S)β1-4GlcUA(2S)β1-3GalNAc(6S) |
|  | ΔD-C | Δ^4,5^HexUA(2S)1-3GalNAc(6S)β1-4GlcUAβ1-3GalNAc(6S) |
|  | ΔC-E | Δ^4,5^HexUA1-3GalNAc(6S)β1-4GlcUAβ1-3GalNAc(4S,6S) |
|  | ΔE-C | Δ^4,5^HexUA1-3GalNAc(6S, 4S)β1-4GlcUAβ1-3GalNAc(6S) |
| Hep/HS tetrasccharide | Δ6S-NS6S | Δ^4,5^HexUA1-4GlcNAc(6S)1-4HexUA1-4GlcNS(6S) |
|  | ΔNS-NS6S  ΔNS6S-NS6S | Δ^4,5^HexUA1-4GlcNS1-4HexUA1-4GlcNS(6S)  Δ^4,5^HexUA1-4GlcNS(6S)1-4HexUA1-4GlcNS(6S) |
|  | Δ2SNS6S-NS6S | Δ^4,5^HexUA(2S)1-4GlcNS(6S)1-4HexUA1-4GlcNS(6S) |
|  | Δ2SNS6S-2SNS6S | Δ^4,5^HexUA(2S)1-4GlcNS(6S)1-4HexUA(2S)1-4GlcNS(6S) |

**Table S4. Strains and primers used in this study.**

|  | Description | | Source |
| --- | --- | --- | --- |
| Strain | |  |  |
| *Bacteroides intestinalis* DSM 17393  *E. coli* BL21(DE3) | | Intestinal microorganisms isolated from human faces  F^-^ *omp*T *hsd*S (rB^-^, mB^-^) *gal dcm* (DE3) | DSMZ |
|  |  |  | Vazyme Biotech. |
| Primers | |  |  |
| GAGase VII-F  GAGase VII-R | | 5’-CATATGCAAGAAGGAGATAG-3’* | Sangon |
|  |  | 5’-CTCGAGGTTATTTTTCCAATC-3’ | Biotech. |

*Restriction enzyme sites are underlined
